# Tachykinin-3 Genes and Peptides Characterized in a Basal Teleost, the European Eel: Evolutionary Perspective and Pituitary Role

**DOI:** 10.3389/fendo.2018.00304

**Published:** 2018-06-11

**Authors:** Aurora Campo, Anne-Gaëlle Lafont, Benjamin Lefranc, Jérôme Leprince, Hervé Tostivint, Nédia Kamech, Sylvie Dufour, Karine Rousseau

**Affiliations:** ^1^Muséum National d’Histoire Naturelle, Research Unit BOREA (Biology of Aquatic Organisms and Ecosystems), CNRS 7208, IRD 207, Sorbonne Université, Université de Caen Normandie, Université des Antilles, Paris, France; ^2^Laboratory of Neuronal and Neuroendocrine Differentiation and Communication, INSERM U1239, Normandy University, Rouen, France; ^3^Muséum National d’Histoire Naturelle, UMR7221 CNRS/MNHN Evolution des Régulations Endocriniennes, Paris, France

**Keywords:** tachykinin-3, neurokinin B, phylogeny, synteny, pituitary cell culture, luteinizing hormone, GnRH-R, teleost

## Abstract

In mammals, neurokinin B (NKB) is a short peptide encoded by the gene *tac3*. It is involved in the brain control of reproduction by stimulating gonadotropin-releasing hormone (GnRH) neurons, mainly *via* kisspeptin. We investigated *tac3* genes and peptides in a basal teleost, the European eel, which shows an atypical blockade of the sexual maturation at a prepubertal stage. Two *tac3* paralogous genes (*tac3a* and *tac3b*) were identified in the eel genome, each encoding two peptides (NKBa or b and NKB-related peptide NKB-RPa or b). Amino acid sequence of eel NKBa is identical to human NKB, and the three others are novel peptide sequences. The four eel peptides present the characteristic C-terminal tachykinin sequence, as well as a similar alpha helix 3D structure. *Tac3* genes were identified *in silico* in 52 species of vertebrates, and a phylogeny analysis was performed on the predicted TAC3 pre-pro-peptide sequences. A synteny analysis was also done to further assess the evolutionary history of *tac3* genes. Duplicated *tac3 g*enes in teleosts likely result from the teleost-specific whole genome duplication (3R). Among teleosts, TAC3b precursor sequences are more divergent than TAC3a, and a loss of *tac3b* gene would have even occurred in some teleost lineages. NKB-RP peptide, encoded beside NKB by *tac3* gene in actinopterygians and basal sarcopterygians, would have been lost in ancestral amniotes. Tissue distribution of eel *tac3a* and *tac3b* mRNAs showed major expression of both transcripts in the brain especially in the diencephalon, as analyzed by specific qPCRs. Human NKB has been tested *in vitro* on primary culture of eel pituitary cells. Human NKB dose-dependently inhibited the expression of *lhβ*, while having no effect on other glycoprotein hormone subunits (*fshβ, tshβ*, and *gpα*) nor on *gh*. Human NKB also dose-dependently inhibited the expression of GnRH receptor (*gnrh-*r2). The four eel peptides have been synthesized and also tested *in vitro*. They all inhibited the expression of both *lhβ* and of *gnrh-r2*. This reveals a potential dual inhibitory role of the four peptides encoded by the two *tac3* genes in eel reproduction, exerted at the pituitary level on both luteinizing hormone and GnRH receptor.

## Introduction

Tachykinins are peptides mainly produced by brain and gut in mammals [for reviews see Ref. (1, 2)]. The most known tachykinin peptides are neurokinin A (NKA), substance P (SP), and neurokinin B (NKB). While NKA and SP are encoded by the *tac1* gene (also named preprotachykinin A gene, PPT-A, or PPT-I), NKB is coded by the *tac3* gene (also named PPT-B or PPT-II, and *tac2* in rodents) [for reviews see Ref. (2–4)]. A second peptide encoded by the *tac3* gene has been recently found in teleosts and was either named neurokinin F (NKF) [zebrafish, *Danio rerio* (5)] or NKB-related peptide (NKB-RP) [grass carp, *Ctenopharyngodon idella* (6); tilapia, *Oreochromis niloticus* (7)]. A *tac4* gene (also named PPT-C or PPT-III) encodes other tachykinins in mammals: hemokinin-1 and endokinins [for reviews see Ref. (3, 4, 8)]. An evolutionary scenario is that an ancestral gene has given rise to four *tac* genes after the two whole genome duplication rounds (1R/2R) in early vertebrates followed by the loss of one of the four paralogs (*tac2*) (9, 10).

Neurokinin B and its receptor in mammals (TACR3, also named NK3 receptor) [for reviews see Ref. (3, 11)] have been involved in the regulation of the gonadotropic axis, after the discovery that mutations of *tac3 or tacr3* genes in humans resulted in a hypogonadotropic hypogonadism (12, 13), reversible in adulthood (14) Similarly, although fertile, *tac3* or *tacr3* null mice exhibited central reproductive defects such as an abnormal estrous cyclicity (15, 16). In addition, *in vivo* studies in different mammals have shown the stimulatory effect of NKB on gonadotropin-releasing hormone (GnRH) and luteinizing hormone (LH) secretion mainly *via* stimulation of the kisspeptin system [mice (17, 18); sheep (19); monkey (20); men and women (21, 22); and rat (23)] [for review see Ref. (24)]. Stimulatory effect of TAC3 on follicle-stimulating hormone (FSH) secretion has also been reported [mice (17, 18); dog (25); monkey (26); and men (22)]. However, a lack of effect or inhibitory action on gonadotropins has also been documented [for reviews see Ref. (27, 28)]. In mammals, NKB is coexpressed with kisspeptin (Kiss) and dynorphin in neurons of the arcuate nucleus of the hypothalamus, which are therefore called KNDy neurons [for review see Ref. (29)]. KNDy neurons project to GnRH neurons and regulate their activity [for reviews see Ref. (24, 27, 28)]. Ablation of KNDy neurons in female rats resulted in hypogonadotropic hypogonadism (30).

In some teleosts, two *tac3* genes have been identified, likely resulting from the teleost-specific whole genome duplication (3R) (10). The role of TAC3 on gonadotropic axis has been studied in some teleosts both *in vivo* and *in vitro*. *In vivo* data showed increase in gonadotropin expression and release after treatment with NKB peptides [zebrafish (5); tilapia (31); goldfish, *Carassius auratus* (32); and orange-spotted grouper, *Epinephelus coioides* (33)]. However, a recent study has also reported the absence of effect of NKB on gonadotropins [tilapia (7)]. *In vitro*, NKB peptides have been shown to be either stimulatory [tilapia (31); striped bass, *Morone saxatilis* (34)], inhibitory [tilapia (7)] or without effect on gonadotropins [grass carp (6); tilapia (7); and orange-spotted grouper (33)]. In contrast to mammals, tachykinin-3 is not coexpressed with kisspeptin in teleost brain [zebrafish (35); striped bass (34)]. However, TAC3 peptides could downregulate *kiss2* expression in striped bass [both NKB and NKB-RP *in vitro* and *in vivo* (34)] and in tilapia [NKB-RP only, *in vivo* (7)], suggesting that tachykinin peptides may act indirectly on GnRH *via* the kisspeptin system, like in some mammals (24, 27). In addition, colocalization of *tacr3* in *lhβ* cells in tilapia pituitary (31) points out that TAC3 peptides as possible direct modulators of gonadotropin secretion in teleosts.

In the European eel, *Anguilla anguilla*, the blockade of the puberty is related to a low stimulation by GnRH (36) and a strong inhibitory control by dopamine (37) [for review see Ref. (38)]. We aimed at investigating TAC3 potential involvement in the control of gonadotropic axis in the European eel. In addition, studies of the tachykinin genes in the European eel, a member of an early group of teleosts (elopomorphs), may provide new insights on ancestral regulations.

In our study, we identified *tac3* genes and encoded peptides in the European eel by *in silico* data mining and cloning. Phylogeny and synteny analyses were performed to infer the molecular evolution of the TAC3 peptides throughout teleost radiation. Tissue distribution of the two eel *tac3* gene expression was investigated by specific qPCRs. The four predicted eel TAC3 peptides were synthesized and tested for their *in vitro* effect on pituitary hormone and receptor expressions by eel pituitary cells.

## Materials and Methods

### Animals

European female eels (*A. anguilla*) were at the prepubertal “silver” stage, corresponding to the end of the continental stage of the eel life cycle, previous to migration to the ocean for reproduction. They were purchased from Gebr. Dil import-export BV (Akersloot, The Netherlands) and transferred to MNHN, France. Animals were anesthetized by cold and then killed by decapitation under the supervision of authorized person (KR; No. R-75UPMC-F1-08) according to the protocol approved by Cuvier Ethic Committee France (No. 68–027).

### *In Silico* Prediction of *tac3* Genes

Tac3 sequences from vertebrate species were retrieved from the Ensembl release 91^1^ and NCBI^2^ databases. Additional blasts were performed using TBLASTN algorithm of the CLC Main Workbench 6 software (QIAGEN Bioinformatics) in the teleost genomes and multiorgan transcriptomes downloaded from NCBI,^3^ Ensembl and Phylofish^4^ (39). The sequences of zebrafish *tac3a* (Gene ID: 100320280) and *tac3b* (Gene ID: 569642) (5) were translated with the EXPASY online tool^5^ and used as queries. The obtained predicted sequences of the tachykinin pre-pro-peptide were added to the query list for a new multiblast in the next species, up to 52 species in total.

For the European eel, blast analyses were performed on both available draft genomes [Illumina (40) and nanopore (41)] and on multiorgan transcriptome [ZF-screens B.V.^6^]. In addition, the draft genomes of the Japanese (*Anguilla japonica*) and American (*Anguilla rostrata*) eels (40, 42) were used for prediction of the *tac3* genes using the European eel transcripts as a query. All sequence references are included in Table S1 in Supplementary Material.

### Cloning and Sequencing of Eel *tac3* cDNAs

The anterior part of the brain including olfactory bulbs (OBs), telencephalon, and di-/mesencephalon was dissected and stored in RNA later (Ambion Inc., Austin, TX, USA) at 4°C (24 h), then at −20°C until extraction. Total RNA was extracted using mechanical homogenization in Trizol Reagent (Invitrogen, Cergy-Pontoise, France), according to the manufacturer’s instructions. Samples were homogenized by TissueLyser II (QIAGEN, Hilden, Germany) and further treated with deoxyribonuclease I (Roche, Meylan, France). RNA quantifications have been performed using a nanodrop spectrophotometer (Thermo Fisher Scientific, Waltham, MA, USA). One microgram of total RNA was reverse-transcribed using SuperScript III First Strand cDNA Synthesis Kit (Invitrogen) and stored at −20°C.

Nested PCRs were performed on anterior brain cDNA using two couples of primers (see Table S2 in Supplementary Material). The primers were designed in the 5′ and 3′ UTRs with Primer 3 online tool (Whitehead Institute/Massachusetts Institute of Technology, Boston, MA, USA) and purchased from Eurofins (Hamburg, Germany). The PCR reactions were carried out on a MyCycler Thermal Cycler (Bio-Rad, Marne-la-Coquette, France) using the GoTaq PCR Core System I (Promega, Charbonnières, France) under the following conditions: 3 min at 94°C; 35 cycles of 1 min at 94°C, 1 min at 2°C under the *T*_m_ of the oligonucleotide with the lowest *T*_m_, and 1 min at 72°C; 7 min at 72°C. The amplification products were subcloned into the pGEM-T Easy vector (Promega) and sequenced (Value Read Sequencing at MWG Biotech, Ebersberg, Germany).

### Phylogeny Analysis

18 Sarcopterygian, 1 chondrichthyan, and 59 actinopterygian TAC3 pre-pro-peptide amino acid sequences were aligned using MUSCLE included in SeaView v. 4.6.1 (43). Alignment was manually adjusted for optimization of key regions such as cleavage sites and signal peptide.

Phylogenic analysis of the TAC3 alignment was achieved using a maximum likelihood method, RAxML black-box^7^ (44), with 1,000 bootstrap replicates and JTT substitution matrix. The TAC3 pre-pro-peptide sequence from the elasmobranchii elephant shark (*Callorhinchus milii*) was chosen as outgroup. The resulting phylogenetic tree was displayed using Figtree v1.4.3. Nodes were collapsed for bootstrapping values below 50% using Mesquite v2.1. Phylogeny analyses were also performed on neighboring genes of *tac3* genomic region: *c1galt1* (core 1 synthase, glycoprotein-*N*-acetylgalactosamine 3-beta-galactosyltransferase 1) and *b4galnt1* (beta-1,4-*N*-acetyl-galactosaminyltransferase 1).

### Synteny Analysis

*Tac3* genomic region of a non-teleost actinopterygian, spotted gar (*Lepisosteus oculatus*) was chosen as reference, using Genomicus PhyloView v91.01.^8^ The genomic regions of eel *tac3a* and *tac3b* were manually analyzed with CLC Main Workbench 6 software on *de novo* assembled European eel draft genome (40, 41). The draft genomes of the Japanese (40) and American (42) eels were also used. *Tac3* neighboring genes were also identified in other representative teleost genomes (golden arowana, *Scleropages formosus*, osteoglossomorph/osteoglossiform; Atlantic herring, *Clupea harengus*, clupeomorph/clupeiform; zebrafish, *D. rerio*, ostariophysi/cypriniform; medaka, *Oryzias latipes*, acanthopterygii/beloniform; tilapia, *Oreochromis niloticus*, acanthopterygii/perciform; stickleback, *Gasterosteus aculeatus*, acanthopterygii/gasterosteiform; fugu, *Takifugu rubipes* acanthopterygii/tetraodontiform) using CLC DNA Workbench for arowana (assembly accession GCF_001624265.1) and herring (GFC 000966335.1) and Genomicus PhyloView for the other species. For each *tac3* neighboring gene family, when only one gene was annotated in all the above teleost mentioned genomes, blast analyses were performed to search for potential additional paralogs.

### Tissue Distribution

Tissues from 10 female silver eels were collected to study the expression of eel *tac3* genes. They were stored in RNA later at 4°C (24 h), then at −20°C, until extraction. The sampled tissues were brain, pituitary, gill, eye, intestine, liver, spleen, and ovary. The brain was dissected in olfactory bulbs, telencephalon, diencephalon, mesencephalon, *cerebellum*, and *medulla oblongata*. Tissue RNA extraction was performed as described in the Cloning and Sequencing section. 500 ng of total RNA were reverse-transcribed using SuperScript III First Strand cDNA Synthesis Kit. Samples were then stored at −20°C until qPCR.

### Prediction and Synthesis of Eel TAC3 Peptides

SignalP 4.1^9^ was used for prediction of signal peptide (45), and Neuropred^10^ for cleavage and amidation sites (46).

Predicted eel TAC3 peptides [NKBa (10 aa), NKB-RPa (13 aa), NKBb (10 aa), and NKB-RPb (13aa)] were synthesized as previously described (47) (Table 1).

**Table 1:** Sequences of European eel (*Anguilla anguilla*) predicted TAC3 peptides.

Gene	Peptide name	Peptide sequence
*tac3a*	NKBa	DMHDFFVGLM-NH_2_
	NKB-RPa	YNGIDYDSFVGLM-NH_2_

*tac3b*	NKBb	DMDDIFVGLM-NH_2_
	NKB-RPb	YNDIDYDTFVGLM-NH_2_

*The predicted sequences are the same for Anguilla japonica and Anguilla rostrata*.

### Prediction of the Three-Dimensional Peptide Structure of Eel TAC3 Peptides

Secondary structures of eel TAC3 peptides were modeled using the I-TASSER server, an automated protein-modeling server (48). Only models with the C-score between 2 and −5 were considered. The visualization of the predicted three-dimensional structures was performed using the RasWin Molecular Graphics software v. 2.7.5.2.^11^

### Pituitary Cell Culture

#### Dispersion and Culture

Dispersion and primary culture of pituitary cells, using 30–40 female eel pituitaries per cell culture experiment, were performed as described in Ref. (49) and as recently used for the test of eel kisspeptins (47). Cultures were performed in serum-free culture medium [M199 with Earle’s salt, sodium bicarbonate, 100 U/ml penicillin, 100 µg/ml streptomycin, and 250 ng/ml fungizone (Gibco, Illkirch, France) at 18°C under 3% CO_2_ and saturated humidity].

#### Treatments

Human NKB (Sigma-Aldrich, Saint-Quentin Fallavier, France) and eel TAC3 peptides stock solutions (10^−4^ M) were prepared in NaOH 0.1 M and stored at −20°C. Stock solutions were diluted in the culture medium just before the addition to the culture wells. The treatments started at day 0 (24 h after cell plating) and five wells per treatment (62,500 cells/well) were used as replicates. Culture medium was changed and peptide solution renewed every 3 or 4 days (day 4 and day 7), and culture was stopped at day 10. The effects of the treatments were tested on three independent experiments performed on cell cultures from different batches of fish and figures display the results of representative experiments. For human NKB, a range of concentrations from 10^−12^ to 10^−6^ M was tested according to previous *in vitro* studies with neuropeptides (47, 50). For synthetized eel NKB and NKB-RP peptides, 10^−7^ M was chosen as submaximal response could be observed with 10^−6^ M human NKB.

#### RNA Extraction and cDNA Synthesis

Total RNA was directly extracted as previously described (47, 50). Briefly, cells were washed with sterile PBS (Gibco) and lysed with Cell-to-cDNA™ II Cell Lysis II Buffer (Ambion; 80 μl/well). The lysates were digested with RNase-free DNase I (Roche). Eight microliters of RNA solution of each sample were then reverse-transcribed with a SuperScript III First Strand cDNA Synthesis Kit (Invitrogen) and stored at −80°C. The cDNA samples obtained were stored at −20°C until qPCR.

### Quantitative Real-Time PCR (qPCR)

#### Primers

Primers for eel *tac3a* and *tac3b* were designed based on sequences of European eel TAC3 pro-peptides using Primer 3 (Table S2 in Supplementary Material) and purchased at Eurofins. Amplicon sizes were 140 bp for *tac3a* and 190 bp for *tac3b*. Forward and reverse primers of each couple were located in different exons to prevent amplification of genomic DNA. To assess the specificity of the qPCR primers, each couple was tested for its inability to amplify the transcript of the other *tac3* gene.

The housekeeping gene was *β-actin* as previously reported (50, 51). Primers for eel *lhβ, fshβ, β subunit of thyroid-stimulating hormone* (*tshβ*), *common α subunit of glycoprotein* (*gpα*), *type 2 GnRH receptor* (*gnrh-r2*), and growth hormone (*gh*) have already been described [Table S2 in Supplementary Material (51–53)]. European eel possesses three GnRH receptors (GnRH-R1a, GnRH-R1b, and GnRH-R2) (53), but *gnrh-r1a* and *gnrh-r1b* expression was below the threshold of detection in cultures of pituitary cells (47), and thus were not assayed in this study.

#### SYBR Green Assay

Quantitative PCR assays were performed using the LightCycler^®^ System (Roche) with SYBR Green I sequence-unspecific detection as previously described (47, 50). Briefly, the qPCRs were prepared with 2 µl of RNase-free water (Ambion), 2 µl of SYBR Green master mix (Roche), 1 µl of each forward and reverse primer (500 nM final concentration), and 4 µl of diluted cDNA template. The protocol was an initial step of polymerase activation for 10 min at 95°C; then 41 cycles (*β-actin, gh, gpα, lhβ, fshβ*, and *tshβ*) or 45 cycles (*tac3a* and *tac3b*) of 10 s at 95°C for denaturing, 5 s at 60°C for annealing, 10 s at 72°C for primer extension and a single final extension step of 5 min at 72°C. For *gnrh-r2*, the protocol was an initial step of polymerase activation for 10 min at 95°C; 42 cycles of 10 s at 95°C, 7 s at 61°C, 4 s at 72°C and a single final extension step of 5 min at 72°C. Each program ended with a melting curve analysis by slowly increasing the temperature (0.01°C/s) from 68 to 95°C with a continuous registration of changes in fluorescent emission intensity. Serial dilutions of cDNA pool of brain (tissue distribution) or pituitary cells (cell culture) were used as a standard curve. One chosen dilution was also included in each run as a calibrator. Normalization of data was performed using total RNA levels (tissue distribution) and *β-actin* mRNA level (cell culture experiments).

### Statistical Analysis

Data are presented as the mean ± SEM. Mean values were compared by Student’s *t*-test or one-way ANOVA followed by Tukey’s multiple comparison test, using Instat (GraphPad Software Inc., San Diego, CA, USA). Differences between groups with *P* < 0.05 were considered statistically significant.

## Results

### Characterization of European Eel *tac3* Genes, Transcripts, and Peptides

#### *In Silico* Identification of Eel *tac3* Genes and Cloning of Transcripts

**Figure 1:**
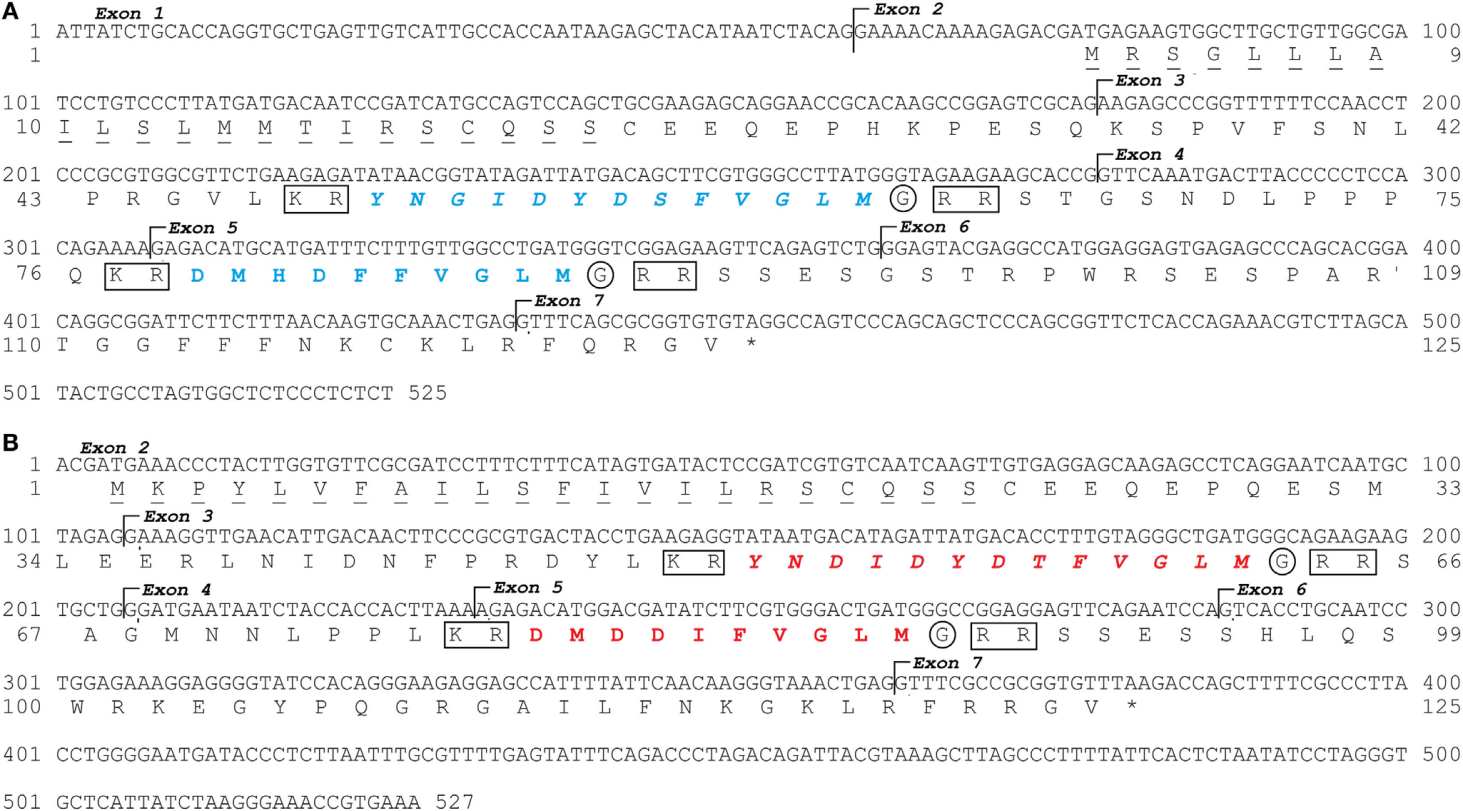
European eel *tac3a* and *tac3b* sequences. Nucleotide and deduced amino acid sequences of *tac3a* **(A)** and *tac3b* **(B)**. Nucleotides (top) are numbered from 5′ to 3′. The amino acid residues (bottom) are numbered beginning with the first methionine residue in the ORF. The asterisk (*) indicates the stop codon. The seven exons are indicated. The amino acids of the signal peptide are underlined. The TAC3 peptides encoded by *tac3a* are in blue and by *tac3b* in red. Related peptides are in italic. The cleavage sites are marked by a square and amidation sites by a circle.

Two *tac3* genes were identified in the European eel genome as well as in the transcriptome (Figure 1). Using European eel specific *tac3a* primers designed on eel *tac3a* predicted genomic sequence, and *tac3b* primers designed on eel *tac3b* predicted genomic sequence (Table S2 in Supplementary Material), PCRs were performed on brain cDNAs. A CDS of 378 bp was characterized for each *tac3* (*tac3a*: MH107060; Figure 1A and *tac3b*: MH107060; Figure 1B). Once translated, both pre-pro-peptide sequences were 125 aa long. BLASTN analyses performed on the European eel draft genome, using the present *tac3a* and *tac3b* cloned sequences as queries, revealed that each transcript is encoded by 7 exons. The pre-pro-peptide is encoded between exons 2 and 7 and the mature peptides by exon 3 (NKB-RP) and exon 5 (NKB) (Figure 1).

#### Prediction of Mature European Eel TAC3 Peptides

Both eel pre-pro-peptide sequences encode two peptides (Figure 1). These peptides were delimited by cleavage sequences at both ends (KR at N-terminal and RR at C-terminal). The C-terminal end of the sequence shows a glycine before the cleavage site, which indicates an amidation site.

The eel NKBa peptide (10 aa) encoded by *tac3a* gene (Figure 1; Table 1) has the same sequence as human NKB. This sequence is also conserved in various sarcopterygians (coelacanth, sauropsids, and mammals) as well as in the non-teleost actinopterygian, spotted gar (Figure S1 in Supplementary Material). By contrast, variations in the sequence are observed in all other teleosts studied (Figure S1 in Supplementary Material). The eel NKB-RPa (13 aa) peptide encoded by *tac3a* gene as well as both peptides, eel NKBb (10 aa) and eel NKB-RPb (13 aa), encoded by *tac3b* gene (Figure 1; Table 1) are novel peptide sequences. These peptides have the same sequences in the three eel species studied (European, American, and Japanese eels) (Table 1; Figure S1 in Supplementary Material).

### Phylogeny Analysis of TAC3

**Figure 2:**
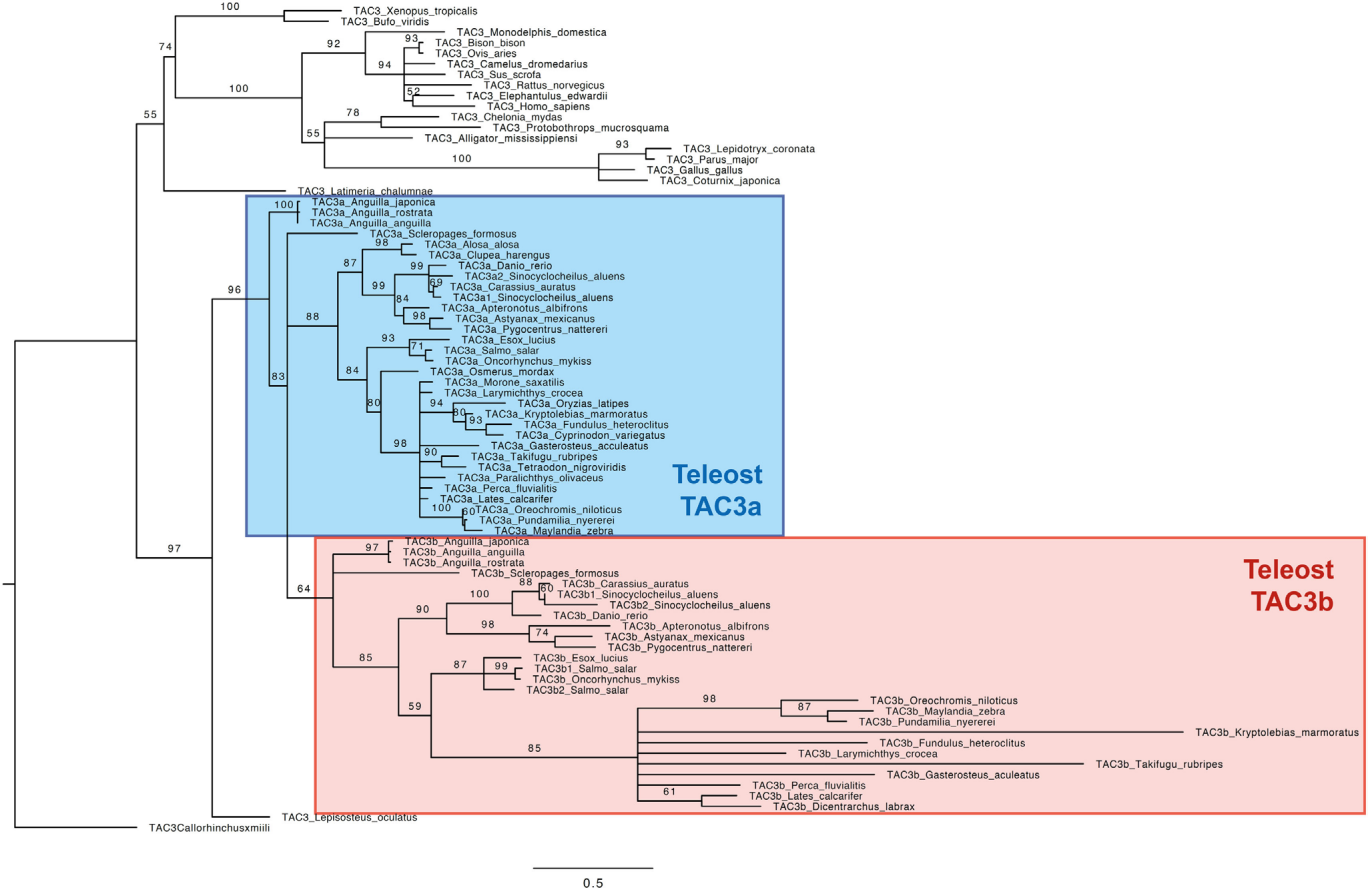
Consensus phylogenetic tree of vertebrate TAC3 pre-pro-peptide amino acid sequences. This phylogenetic tree was based on the deduced amino acid sequences of *tac3* (see Table S1 and Figure S1 in Supplementary Material) using the maximum likelihood method with 1,000 bootstrap replicates. The number shown at each branch node indicates the bootstrap value (%); only values and branching above 50% are indicated. The tree was rooted using the TAC3 sequence of the elephant shark *Callorhinchus milii*. The teleost TAC3a and TAC3b sequences are colored in blue and red, respectively.

Based on an alignment of 78 TAC3 pre-pro-peptidic amino acid sequences (Figure S1 in Supplementary Material), and assuming the elephant shark *C. milii* sequence as outgroup, a phylogenetic tree was generated using the maximum likelihood method (the list of sequences and accession numbers is provided in Table S1 in Supplementary Material) (Figure 2).

As shown in Figure 2, the TAC3 pre-propeptide sequences are clustered into two main clades: sarcopterygians and actinopterygians. In the sarcopterygian clade, TAC3 sequences of birds have diverged, as indicated by the long branch of this group. The spotted gar TAC3 sequence branches at the base of the actinopterygian clade. In most teleosts, two TAC3 (a and b) were found, TAC3a being present in all investigated species, while TAC3b not being retrieved in a few species even with available genome such as a clupeomorph (herring), and an acanthopterygii (medaka). Teleost TAC3b sequences form a single clade, including elopomorph (eel) and osteoglossomorph (arowana) sequences branching at its basis. By contrast, a well-supported clade is observed only for clupeocephala TAC3a sequences. Basal teleost TAC3a sequences are not included in this clade: eel TAC3a sequences branch at the basis of all teleost TAC3a and TAC3b sequences, and arowana TAC3a is in polytomy with clupeocephala TAC3a and teleost TAC3b. From this phylogenetic analysis, we could suggest that the two teleost *Tac3* genes likely resulted from 3R, but the classification of the basal teleost TAC3 sequences needed to be further assessed by synteny analysis.

### Synteny Analysis of *tac3* Genomic Region

**Figure 3:**
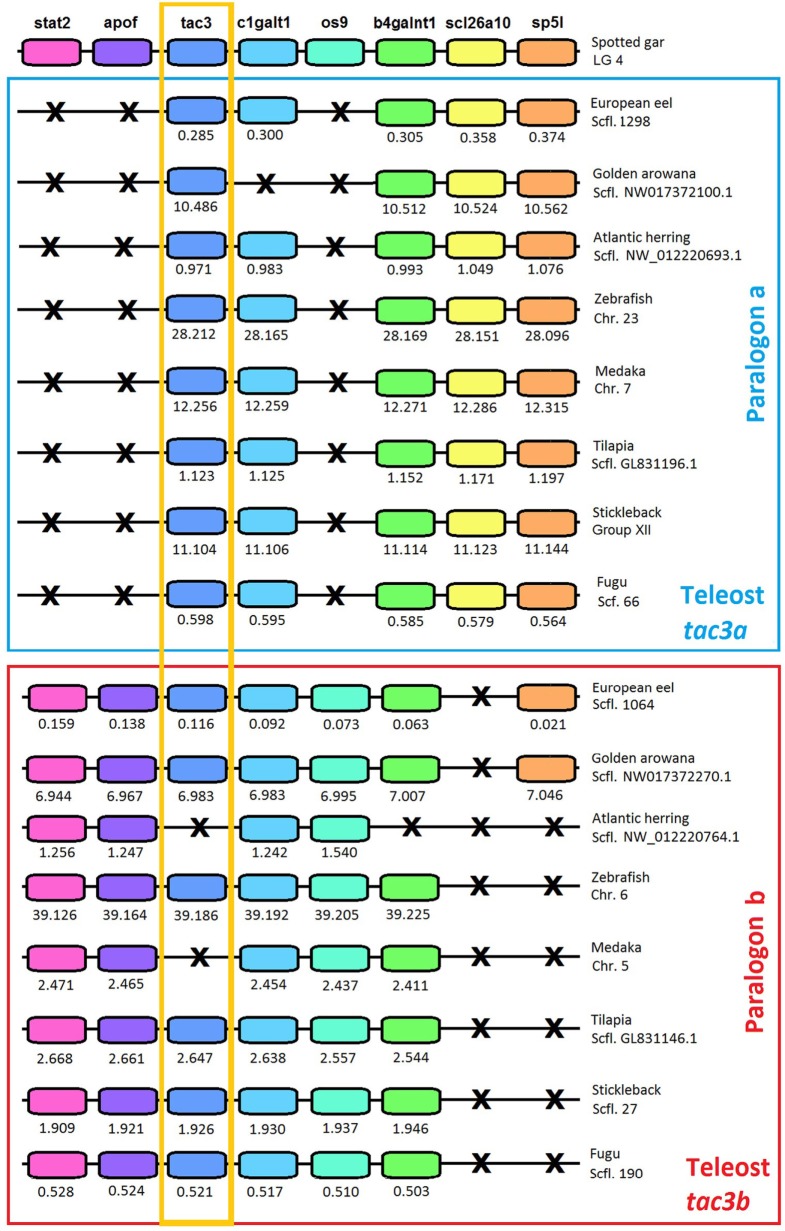
Syntenic region of *tac3* in key teleost species and the actinopterygian spotted gar. Spotted gar *tac3* genomic region is used as a reference. This region is duplicated in teleosts. Blue square includes teleost paralogon a, and red square includes teleost paralogon b. Gene color is preserved for homologous genes. The *tac3* genes are highlighted by an orange square. Gene losses are marked with a cross. For each species, chromosome or scaffold is indicated. Below each gene, its position is indicated in 10^6^ bp. The full gene names, reference, and detailed genomic locations are given in Table S3 in Supplementary Material.

To further resolve the origin and nomenclature of the duplicated eel *tac3a* and *tac3b*, we performed a synteny analysis (Figure 3) on the *tac3* genomic region of representative species of various teleost superorders: European eel (elopomorph), golden arowana (osteoglossomorph), Atlantic herring (clupeomorph), zebrafish (ostariophysi), medaka, tilapia, stickleback, and fugu (acanthopterygii). The spotted gar, a non-teleost actinopterygian, was chosen as the reference species in this synteny analysis. In all teleosts, the *tac3* genomic region was duplicated in agreement with the 3R (Figure 3). For *tac3*, synteny analysis highlights the loss for *tac3b* in some species such as herring and medaka. As these two species belong to different teleost superorders/orders, this indicates independent recurrent events of *tac3b* loss throughout teleost radiation: at least in the clupeomorph/clupeiform lineage and in the acanthopterygii/beloniform lineage. Some *tac3* neighboring genes 3R-paralogs were conserved in all studied teleosts: *c1galt1* (except in golden arowana) and *b4galnt1*. By contrast, for other *tac3* neighboring genes, only a single 3R-paralog was conserved. For instance, *scl26a10* was conserved on *tac3a* paralogon, but lost on *tac3b* paralogon, including in eel and arowana. Conversely, 3R-paralogs of *stat2, apof*, and *os9* were conserved on *tac3b* paralogon and lost on *tac3a* paralogon, including in eel and arowana. These syntenic data allow us to definitely assign eel and arowana duplicated *tac3* genes as *tac3a* and *tac3b*, respectively.

To further assess these orthologies, we also performed phylogeny analyses on *tac3* neighboring genes, *c1galt1* (Figure S3 in Supplementary Material) and *b4galnt1* (Figure S5 in Supplementary Material), the 3R-duplicated paralogs of which have been conserved in most teleosts. For *c1galt1* (Figure S3 in Supplementary Material), teleosts sequences located on paralogon a did not group into a clade’; however, a well-supported clade grouped all teleost sequences located on paralogon b, including those of eel and arowana branching at the basis of this clade. This phylogeny is in agreement with the respective assignment of eel and arowana *tac3* sequences to paralogons a and b. For *b4galnt1* (Figure S5 in Supplementary Material), all actinopterygian sequences formed a well-supported clade with the single spotted gar sequences branching at its basis. Teleosts 3R-*b4galnt1* duplicated paralogs split into two clades, each one encompassing eel and arowana sequences. Each teleost clade corresponded to the 3R-duplicated genes located on the respective *tac3a* and *b* paralogons. This phylogeny fully supported the respective assignment of eel and arowana *tac3* sequences to paralogons a and b.

### Tissue Distribution of Eel *tac3a* and *tac3b* Transcripts

**Figure 4:**
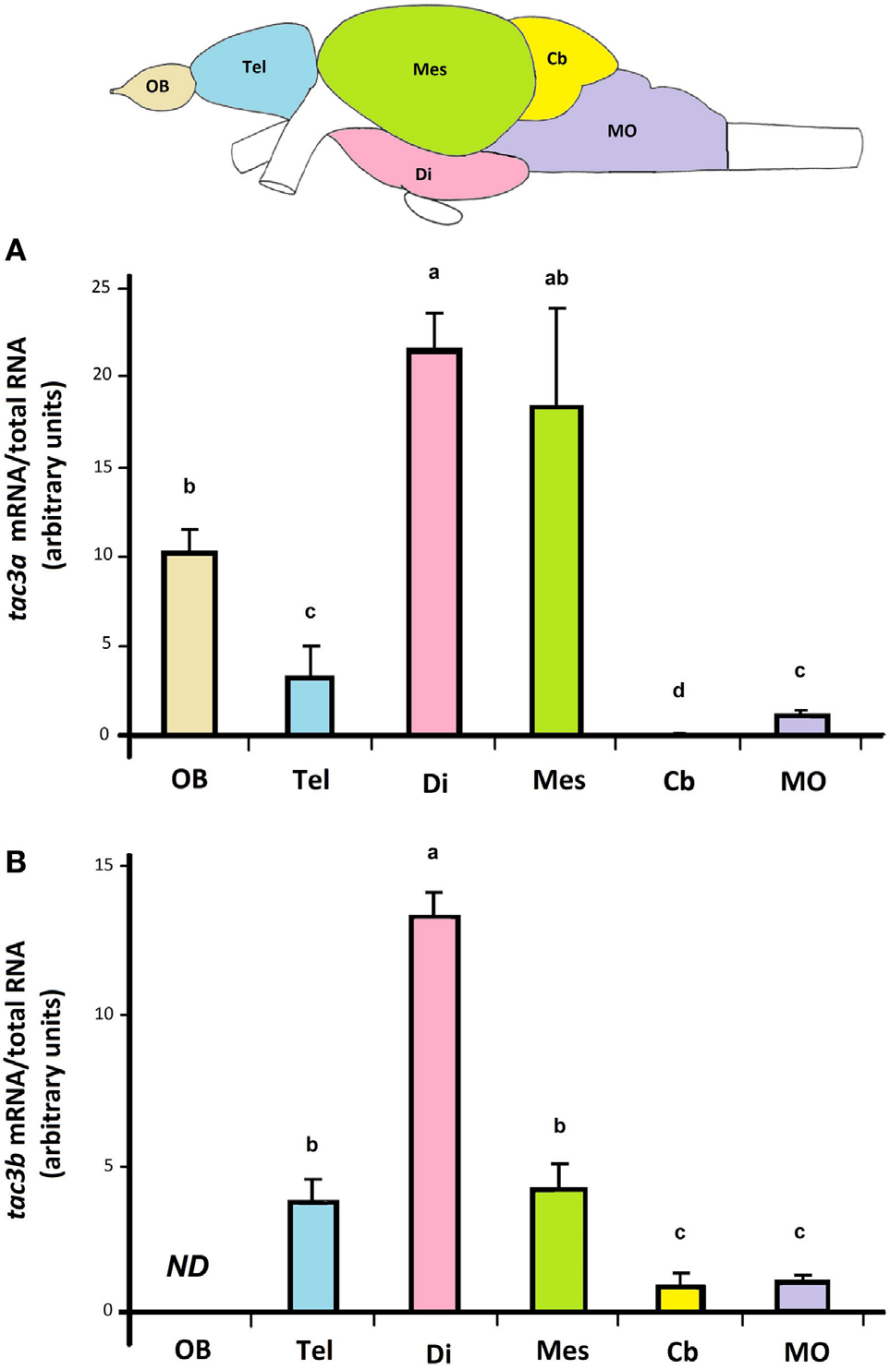
Tissue distribution of eel *tac3a* and *tac3b* transcripts. Olfactory bulb (OB), telencephalon (Tel), diencephalon (Di), mesencephalon (Mes), *cerebellum* (Cb), and *medulla oblongata* (MO) were dissected from prepubertal female eels. The expression of *tac3a* mRNA **(A)** and of *tac3b* mRNA **(B)** were measured by qPCRs in each brain region and normalized to total RNA. Each bar represents the mean ± SEM from 10 individual samples. Different letters denote statistical significance (*P* < 0.05). Abbreviation: ND, non detectable.

Both *tac3a* and *tac3b* transcripts were mainly expressed in the brain. Figure 4 displays expression of *tac3a* and *tac3b* transcripts in different regions of the eel brain. Eel *tac3a* and *tac3b* mRNAs were both predominantly expressed in the diencephalon. *Tac3a* was also highly expressed in the mesencephalon, with weaker levels in OBs, telencephalon and MO. In addition to diencephalon, *tac3b* was expressed to a lesser extent in the mesencephalon and telencephalon, with weak levels in Cb and MO and undetectable levels in OBs.

Concerning peripheral tissues, *tac3a* was weakly expressed in the pituitary and ovary, while *tac3b* was undetectable. Both *tac3a* and *b* were weakly expressed in the intestine, and not detectable in the other tissues investigated (liver, spleen, eye, and gills) (data not shown).

### *In Vitro* Effect of Human NKB and Eel TAC3 Peptides on Pituitary Hormone and *gnrh-r2* Expression by Eel Pituitary Cells

The effects of commercial human NKB and synthesized eel TAC3 peptides were tested over 10 days in eel pituitary cell culture system as previously described for kisspeptins (47).

#### Effects of Human NKB on Pituitary Hormone and *gnrh-r2* Expression

**Figure 5:**
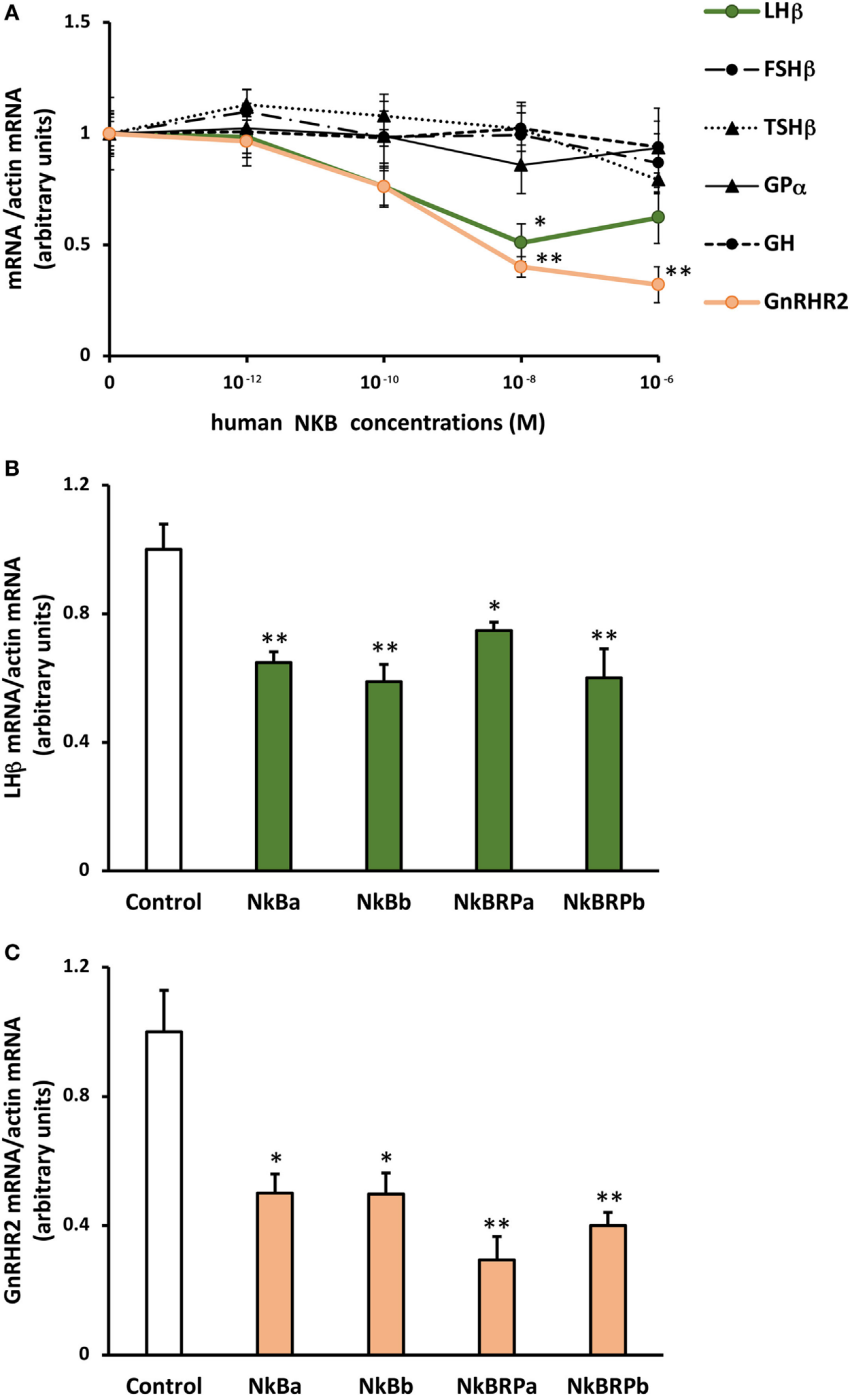
Effect of human **(A)** and eel **(B)** TAC3 peptides on pituitary hormone and *gnrh* receptor expression by eel pituitary cells. **(A)** Primary cultures of pituitary cells were treated with various concentrations (10^−12^, 10^−10^, 10^−8^, and 10^−6^ M) of human neurokinin B (NKB) for 10 days. Pituitary hormones and receptor mRNA levels were quantified by qPCR. **(B,C)** Pituitary cells were treated with 10^−7^ M of eel TAC3 peptides, NKBa, NKBb, NKB-RPa, and NKB-RPb for 10 days. *lhβ* **(B)** and *gnrh-r2* **(C)** mRNA levels were quantified by qPCR. This figure displays the results of representative experiment. Data were normalized against β*-*actin. Each point represents mean ± SEM from five well replicates. *P < 0.05 and **P < 0.01 versus controls, *t*-test or ANOVA.

Human NKB peptide dose-dependently inhibited *lhβ* expression. By contrast, this peptide had no significant effect on the expression of the other glycoprotein hormone subunits (*fshβ, tshβ*, and *gpα*) nor on *gh* (Figure 5A). Human NKB peptide also dose-dependently inhibited *gnrh-r2* expression (Figure 5A).

#### Effects of Eel TAC3 Peptides on *lhβ* and *gnrh-r2* Expression

All four eel synthesized TAC3 peptides (NKBa, NKBb, NKB-RPa, and NKB-RPb), as tested at 10^−7^ M, significantly inhibited *lhβ* expression (Figure 5B).

A significant inhibitory effect of all four synthesized eel NKB peptides at 10^−7^ M was also observed on the expression of *gnrh-r2* (Figure 5C).

### Prediction of the Three-Dimensional Peptide Structure of Eel TAC3 Peptides

**Figure 6:**
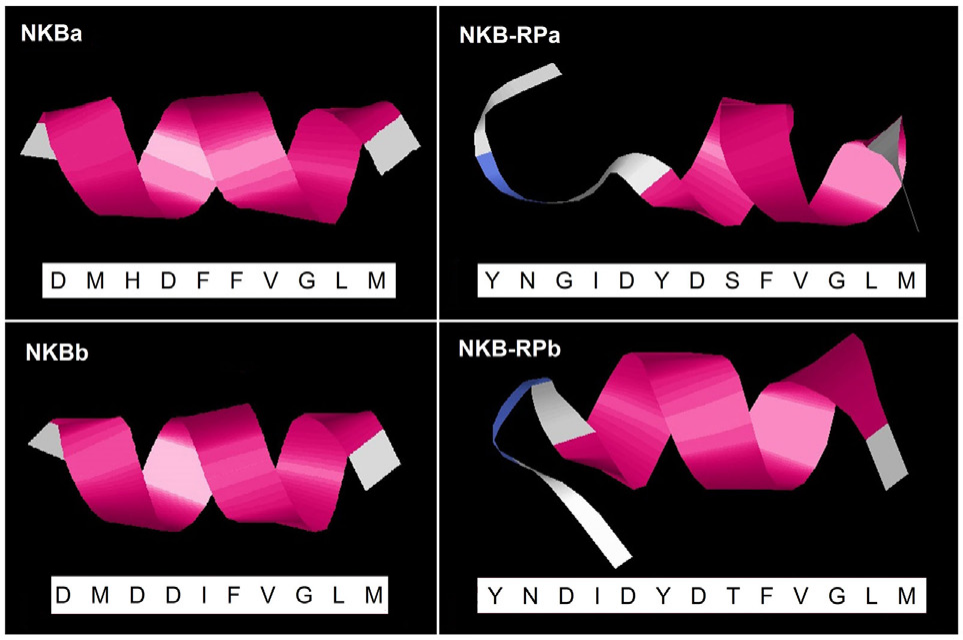
Predicted 3D structure of eel TAC3 peptides. The amino acid sequences of eel NKBa, NKB-RPa, NKBb, and NKB-RPb are indicated. Eel NKBa has the same sequence as human neurokinin B (NKB). Models predicted in I-TASSER server with a *C*-score between 2 and −5 were presented. The C-terminal is oriented toward the right. In pink appears α-helix, in blue turn, and in white random coil.

Predicted secondary structures of eel TAC3 peptides were obtained using the I-TASSER server. As described above, eel NKBa peptide sequence is the same as human NKB. Human NKB 3D structure was already reported [PDB ID 1p9f (5)]. For all four eel peptides, the 3D structure was characterized by a single α-helix, as for human NKB (Figure 6). Random coil and turn structure appear in the N-terminal of the related peptides NKB-RPa and NKB-RPb (Figure 6).

## Discussion

### Two *tac3* Genes and Four TAC3 Peptides in the Eel

We show that the European eel, as well as other eel species, possesses two *tac3* genes, each of them encoding two peptides. *Tac3a* and *tac3b* genes consist of seven exons as human and rat *tac3* genes (54). The sequences that encode NKB (a or b) are located in exon 5, as in human and rat (54), while exon 3 contains the sequence encoding related peptide (NKB-RPa or b), which has been lost in mammals (55).

Among the four eel TAC3 peptides, NKBa has the same amino acid sequence as human NKB. This sequence, which is also identical in various sarcopterygians (coelacanth, some amphibians, sauropsids, and mammals) as well as in a non-teleost actinopterygian, spotted gar, may represent an ancestral NKB sequence largely conserved throughout vertebrate radiation. This sequence still conserved in the eel, a basal teleost (elopomorph) shows variations in the other teleosts, including another basal representative, the arowana (osteoglossomorph) (Figure S1 in Supplementary Material).

The other three eel peptides are totally new peptides: NKBb and the two neurokinin related peptides (NKB-RPa and NKB-RPb). NKB-RP would be present in amphibians but lacking in reptiles, birds and mammals (2, 5, 55), suggesting a loss of NKB-RP in the amniote lineage. In our study, we show that these related peptides were preserved in actinopterygians, in agreement with previous studies in teleosts (5, 7, 10, 31, 32, 34).

Eel TAC3 peptides all showed the characteristic C-terminal signature motif of FxGLM of tachykinin family, where x is a valine [for review see Ref. (3)]. This C-terminal sequence is critical for receptor binding and bioactivities (54). However, in some other teleosts, this C-terminal region of *tac3b* products is more divergent [FVGLL for zebrafish, goldfish, grass carp, and salmon NKBb; LAALL for sea bass NKBb; FIGLM for goldfish and grass carp NKB-RPb; for review see Ref. (2)].

The structural organization of one precursor containing two peptides is conserved within the human TAC1 and TAC4 precursors but differs from that of human TAC3 precursor, where NKB-RP is missing. In *Ciona intestinalis*, two tachykinin-like peptides are also produced by one *tac* gene (56), suggesting that the co-existence of two tachykinin peptides within a single precursor is the ancestral organization of TAC precursors [for review see Ref. (10)].

The 3D model structures of all four eel TAC3 peptides shows a α-helix structure, as previously reported for human NKB as well as for zebrafish TAC3 peptides (5). This helical structure was demonstrated for mammalian NKB in the presence of dodecyl phosphocholine (57) and sodium dodecyl sulfate (58) micelles. In mammals, the formation of a helical conformation in the mid region of TAC3 peptide has been shown to be crucial for tachykinin receptor activation (54). This structure would thus provide to eel TAC3 peptides a binding-competent conformation similar to that of human NKB.

### Eel Duplicated *tac3* Genes Come From 3R

Two *tac3* genes are present in the eel, as in most other teleost species investigated, while only a single gene is present in the non-teleost actinopterygian, spotted gar, and in sarcopterygian species. These two paralogs in teleosts likely result from the teleost-specific whole genome duplication (3R). Phylogeny analysis showed that teleost pre-pro-TAC3a amino acid sequences are relatively conserved, while pre-pro-TAC3b sequences have largely diverged in some species. A loss of *tac3b* would have even happened, independently in some teleost lineages, such as in clupeiform (herring) and beloniform (medaka) as shown herein. An independent loss of *tac3b* could have also occurred in other teleost subgroups, as suggested by Chen et al. (33) for the grouper (perciform, serranidae).

The TAC3 phylogeny analysis conducted in this study was not informative enough to allow us to unequivocally classify the two eel TAC3 into the teleost TAC3a and TAC3b. The situation was the same for the two TAC3 from arowana, another representative of basal teleosts. Toward this aim, we performed synteny analysis of *tac3* genomic regions in representative species of teleosts, and using a non-teleost actinopterygian, spotted gar, as a reference. The synteny analysis supports that the whole genomic region containing *tac3* would have been duplicated in teleosts, probably as a result of 3R. Concerning *tac3*, our syntenic analysis allowed us to clearly classify the two eel, as well as the two arowana, genes. We considered the eel and arowana *tac3* located in the same paralogon as *scl26a10* as orthologs to teleost *tac3a*, and the eel and arowana *tac3* located in the same paralogon as *stat2, apof, os9* as orthologs to teleost *tac3b*. Phylogeny analyses of duplicated neighboring genes also supported this conclusion. The two eel *tac3* were therefore named accordingly, eel *tac3a* and *tac3b*. Our synteny data strengthens the hypothesis of the 3R origin of the two *tac3* genes in the eel as in most other teleosts, with the loss of *tac3b* in some teleost species representing lineage specific events.

### Eel *tac3a* and *tac3b* Are Mostly Expressed in the Brain

Both eel *tac3* mRNAs were mainly expressed in the diencephalon. This brain region is the major neuroendocrine region of the brain in vertebrates. In mammals, it is where the KNDy neurons are localized [for reviews see Ref. (2, 59)]. Eel *tac3a* and *b* expressions were also observed in other parts of the brain. Apart from the diencephalon, *tac3a* gene expression was expressed in the OB and mesencephalon, while the *tac3b* was expressed in telencephalon and mesencephalon.

A wide brain expression was also observed in other teleosts. In zebrafish, *tac3a* cerebral expression was observed either mainly in “midbrain” (including optic tectum/mesencephalon-diencephalon and hypothalamus) (5, 35) or predominantly in hypothalamus with low levels in telencephalon and optic tectum-thalamus (10). In this species, *tac3b* mRNA was mainly expressed in “forebrain” (telencephalon) (5, 10, 35) as well as in hypothalamus (10). In goldfish, both *tac3a* and *tac3b* mRNA were found in telencephalon, optic tectum-thalamus, Cb and hypothalamus (32). In grass carp, the expression of the single *tac3* (*tac3a*) was mostly observed in the OB and hypothalamus (6). Using *in situ* hybridization, a widespread distribution of *tac3* expression was reported in the brain of zebrafish (5, 35), goldfish (32), striped bass (34) and orange-spotted grouper (33). In mammals (human, rat and mouse), a large brain distribution of *tac3* transcripts has also been reported, the expression not being restricted to KNDy neurons [for reviews see Ref. (1, 59)].

In the pituitary, a weak expression of *tac3a* but no detectable expression of *tac3b* was observed in the eel. This differential expression was also reported in goldfish (32). In some species, where only *tac3a* has been identified, a weak expression was found in the pituitary [orange-spotted grouper (33); grass carp (6)]. By contrast, in zebrafish (5, 35), both genes were found to be expressed in the pituitary. Pituitary expression of *tac3* is also observed in mammals (54).

In the ovary, weak expression of *tac3a* (and none for *tac3b*) was found in the eel, as in goldfish (32) and orange-spotted grouper (33). In zebrafish, contradictory results were obtained with sexually mature fish: while *tac3b* gene was found to be expressed in the ovary according to some authors (5, 10), it was *tac3a* gene in another study (35). Mammalian *tac3* gene expression is found in reproductive organs such as placenta, uterus, ovary, oviduct, prostate gland, and testis [for review see Ref. (2)]. NKB was reported to be important in normal follicle growth as well as in estradiol preovulatory and progesterone postovulatory rise in women (21). Direct role of NKB on estradiol production has been recently observed in zebrafish primary cultures of follicular cells and in human cell line derived from a granulosa tumor (60).

A weak expression of both *tac3a* and *b* was detected in the intestine of the eel. This tissue expressed *tac3a* (and not *tac3b*) in zebrafish and goldfish (32), and the single *tac3* gene (*tac3a*) in grass carp (6) and orange-spotted grouper (33). In mammals, while works described the localization of NKB in intestine, no study has yet provided evidence for the existence of *tac3* gene at this level [for reviews see Ref. (1, 61)]. However, NK3 receptor expression is observed in the gastrointestinal tract and NKB has been shown to induce contractile responses [for review see Ref. (54)].

### Eel TAC3 Peptides Exert a Dual Inhibitory Effect on Pituitary Gonadotropic Function

In this study, we showed that human NKB as well as all four synthetized eel TAC3 peptides (NKBa, NKBb, NKB-RPa, and NKB-RPb) were able to regulate hormone and receptor expression by eel pituitary cells in culture. This reveals a direct pituitary effect of TAC3 peptides in the eel.

All tested TAC3 peptides inhibited *lhβ* expression, without affecting *fshβ* transcripts. Studies of the *in vitro* effect of TAC3 peptides in other teleost species concerned only peptides encoded by *tac3a* gene and named below NKB and NKB-RP/NKF. They revealed variable effects on gonadotropins. In striped bass, NKB and NKB-RP (NKF) enhanced LH and FSH releases by primary pituitary cultures, and no effect was observed on *lhβ* and *fshβ* transcript levels (34). In tilapia, NKB and NKB-RP (NKF) induced LH and FSH release by primary cultures of mature male pituitary cells (31), in agreement with the presence of NKB receptors (*tac3ra* and *tac3rb*) on both cell types (31). By contrast, in the same species, NKB-RP could decrease *lhβ* and *fshβ* expression by pituitaries of juvenile mixed-sex animals with no effect of NKB (7). In grass carp, Hu and collaborators (6) reported no variation of LH and FSH expression, cell content and release after NKB or NKB-RP treatment of pituitary cells. Similarly, orange-spotted grouper NKB and NKB-RP had no effect on gonadotropin mRNA levels in cultured pituitary cells (33). As far as one can tell from the literature, our study is the first to investigate the *in vitro* effect of TAC3 peptides encoded by both *tac3a* and *tac3b* genes in teleosts. We show that these peptides are all able to inhibit *lhβ* expression in the prepubertal female eel. To the best of our knowledge, only one *in vitro* study has been reported in mammals. Using gonadotroph cell line LβT2, Mijiddorj and collaborators observed no effect of NKB on *lhβ* and *fshβ* mRNA expression, albeit NKB receptor was detected (62).

When administered *in vivo* in teleosts, NKB peptides could either increase [zebrafish (5); tilapia (31); and goldfish (32); NKB in orange-spotted grouper (33)] or had no effect [tilapia (7); NKB-RP in orange-spotted grouper (33)] on gonadotropin expression and/or release. Comparing various tachykinins *in vivo*, Sahu and Kalra (63) were the first to report that NKB-containing implants, in the third ventricle of ovariectomized rat brain, did not induce any change in LH release. Later, this absence of NKB effect on LH was also shown after either intraperitoneal or intracerebroventricular administration to male mice (64). In this last study, NKB was even ineffective in stimulating GnRH secretion by hypothalamic rat explants (64). However, evidence for stimulatory [mouse (17, 18); monkey (20, 26, 65); sheep (19, 26, 66–68); and human (21, 22)] or inhibitory [rat (69); mouse (70); and goat (71)] effects of NKB on LH have also been documented. Concerning FSH, either stimulatory [mouse (17, 18); dog (25); monkey (26); and man (22)] or no effect [mouse (64); women (21)] of NKB has been reported. These discrepancies in mammals as in teleosts could be due to species, physiological status, or mode of peptide administration.

The downregulation of *gnrh-r2* expression that we observed in our study after treatment of eel pituitary cells by commercial human NKB and synthesized eel TAC3 peptides has never been reported before. We have also recently demonstrated a decrease of *gnrh-r2* expression after kisspeptin treatment by eel pituitary cells (47), in parallel to a decrease of *lhβ* expression. These results suggest that a double inhibitory control could be exerted by different neuropeptides on pituitary gonadotropic function: by downregulating *lhβ* expression and by decreasing pituitary sensitivity to GnRH *via* downregulation of *GnRH* receptor expression. This is in good agreement with the low expression levels of *gnrh-r2* in the pituitaries of males and females at the silver stage (53). The neurokinin and kisspeptin systems may thus contribute to the strong inhibitory control of eel reproductive function.

Contrary to the downregulation of both *lhβ* and *gnrh-r2* expression, we observed no regulation of the expression of other pituitary hormones (*fshβ, tshβ*, and *gh*) by eel pituitary cells after treatment with human and eel TAC3 peptides. In carp, homologous NKB and NKB-RP did not affect *gh, lhβ, fshβ, tshβ, somatolactin β* (*slβ*), *pomc*, and *gpα* expression, nor GH, LH and SLβ release, by primary cultures of pituitary cells, but they did induce secretion, cell content and mRNAs of prolactin (PRL) and SLα (6). These *in vitro* results in the carp were in agreement with hybridization signals for neurokinin receptors, NK2 receptors on PRL cells, NK3 receptors on SLα cells, with absence of neurokinin receptor signals in other cell types, SLβ and GH cells (6). In mammals, such stimulatory role of NKB has already been reported on PRL release in rat pituitary cells (72) and on TRH-induced *prl* mRNA expression in somatolactotroph GH3 cell line (62). Future studies should aim at investigating other pituitary hormones (such as PRL and SL) and localization of TAC3R receptors in eel pituitary.

In this study, the four eel TAC3 peptides have a consistent effect: inhibition of *lhβ* and *gnrh-r2* expression. The biological activity and similar effect of these peptides may be related to their characteristic C-terminal tachykinin motif, and their conserved 3D α-helix structure. This may indicate a system of tachykinergic co-transmission to modulate the response, as suggested for TAC1 peptides (73). In this way, the effect of one peptide may be modulated by the co-expression of all other three, thus adjusting the response to the possible different signals.

This study addressed the effects of TAC3 peptides on pituitary gonadotropin and *gnrh-r* expression. Future studies may aim at investigating the possible action of TAC3 peptides on kisspeptin and kisspeptin receptors (47, 74) as well as dopamine receptors (75, 76) to further decipher TAC3 mechanisms of actions and interactions at the pituitary level. Potential interactions between tachykinin, kisspeptin, GnRH, and dopamine systems remain also to be explored at the brain level.

In conclusion, in a basal teleost, the European eel, we identified two *tac3* genes encoding four TAC3 peptides, NKBa which is identical to human NKB, NKB-RPa, NKBb, and NKB-RPb. Phylogeny and synteny analyses allowed us to infer that these two genes likely result from teleost-specific whole genome duplication (3R). The two paralogous genes *tac3a* and *tac3b* have been conserved in most teleost species, but large sequence divergence is observed for *tac3b* and recurrent events of loss of *tac3b* paralog have occurred independently in some teleost lineages. In the eel, the two *tac3* are mainly expressed in the brain, with high levels in the diencephalon known to contain hypophysiotropic neurons. Concerning the pituitary role of the TAC3 eel peptides, our study demonstrates for the first time *in vitro* effects of NKBb and its related peptide. The four peptides present in the European eel are able to downregulate *lhβ* and *gnrh-r2* transcripts in primary cultures of eel pituitary cells. Thus, in the eel, NKB peptides exert a double inhibitory control on gonadotropic function, by decreasing *lhβ* expression directly at the pituitary level, and also by reducing pituitary sensitivity to GnRH *via* downregulation of *GnRH* receptor expression. The tachykinin system, as previously shown with the kisspeptin system, may thus contribute to the strong inhibitory control of puberty observed in the European eel.

## Ethics Statement

Animals were anesthetized by cold and then killed by decapitation under the supervision of authorized person (KR; No. R-75UPMC-F1-08) according to the protocol approved by Cuvier Ethic Committee France (No. 68-027).

## Author Contributions

AC and HT: cloning. AC, A-GL, and SD: phylogeny and synteny analyses. BL and JL: synthesis of eel TAC3 peptides. AC and NK: 3D prediction. KR: test of peptides on primary cultures. AC: qPCR. KR and SD: design of the experiments. KR, SD, A-GL, and AC: writing of the manuscript. All the authors approved the final version of the manuscript.

## Conflict of Interest Statement

The authors declare that the research was conducted in the absence of any commercial or financial relationships that could be construed as a potential conflict of interest.

## Footnotes

^1^http://www.ensembl.org/index.html.

^2^https://www.ncbi.nlm.nih.gov/.

^3^ftp://ftp.ncbi.nlm.nih.gov/genomes/.

^4^http://phylofish.sigenae.org/index.html.

^5^https://web.expasy.org/translate/.

^6^https://drive.google.com/open?id=1-GfA0fo0xOz7hRc_bOYs9ozek2KKd0zK, https://drive.google.com/open?id=1YcrkFNy-AIG7WaFsILhs3egHZBvh5VHV, https://drive.google.com/open?id=1df2QsMugOfEyfwLq4OIeXnosqNh3HlDJ, https://drive.google.com/open?id=1dWdlAfP6GI7piXM9h_OPu1ITjRrq7x77, https://drive.google.com/open?id=1QWpkkt_WFidqlLNZDBFjaDeFCtMrze8O, https://drive.google.com/file/d/1onKx-ciZbj_PnQgRwAvX2V5Jvwgvbxqr/view?usp=sharing.

^7^https://www.phylo.org/.

^8^http://www.genomicus.biologie.ens.fr/genomicus-91.01/cgi-bin/search.pl.

^9^http://www.cbs.dtu.dk/services/SignalP/.

^10^http://stagbeetle.animal.uiuc.edu/cgi-bin/neuropred.py.

^11^http://www.rasmol.org/.
