# Supplementary material for: Tachykinin-3 Genes and Peptides Characterized in a Basal Teleost, the European Eel: Evolutionary Perspective and Pituitary Role
Source: Front Endocrinol (Lausanne). 2018 Jun 11;9:304. doi: 10.3389/fendo.2018.00304 (PMC6004781; doi:10.3389/fendo.2018.00304)
Supplement: Supplementary file 1 [file Presentation_1.PDF]

*Supplementary Material*

# TACHYKININ-3 GENES AND PEPTIDES CHARACTERIZED IN A BASAL TELEOST, THE EUROPEAN EEL: EVOLUTIONARY PERSPECTIVE AND PITUITARY ROLE

Aurora Campo, Anne-Gaëlle Lafont, Benjamin Lefranc, Jérome Leprince, Hervé Tostivint, Nédia Kamech, Sylvie Dufour and Karine Rousseau

Correspondence: Karine Rousseau: karine.rousseau@mnhn.fr

## 1. Supplementary Figures

**Supplementary Figure 1.** Alignment of 78 TAC3 pre-pro-peptide sequences used in the phylogeny analysis (Fig 2). The amino-acid residues with similar physico-chemical properties are in the same color.

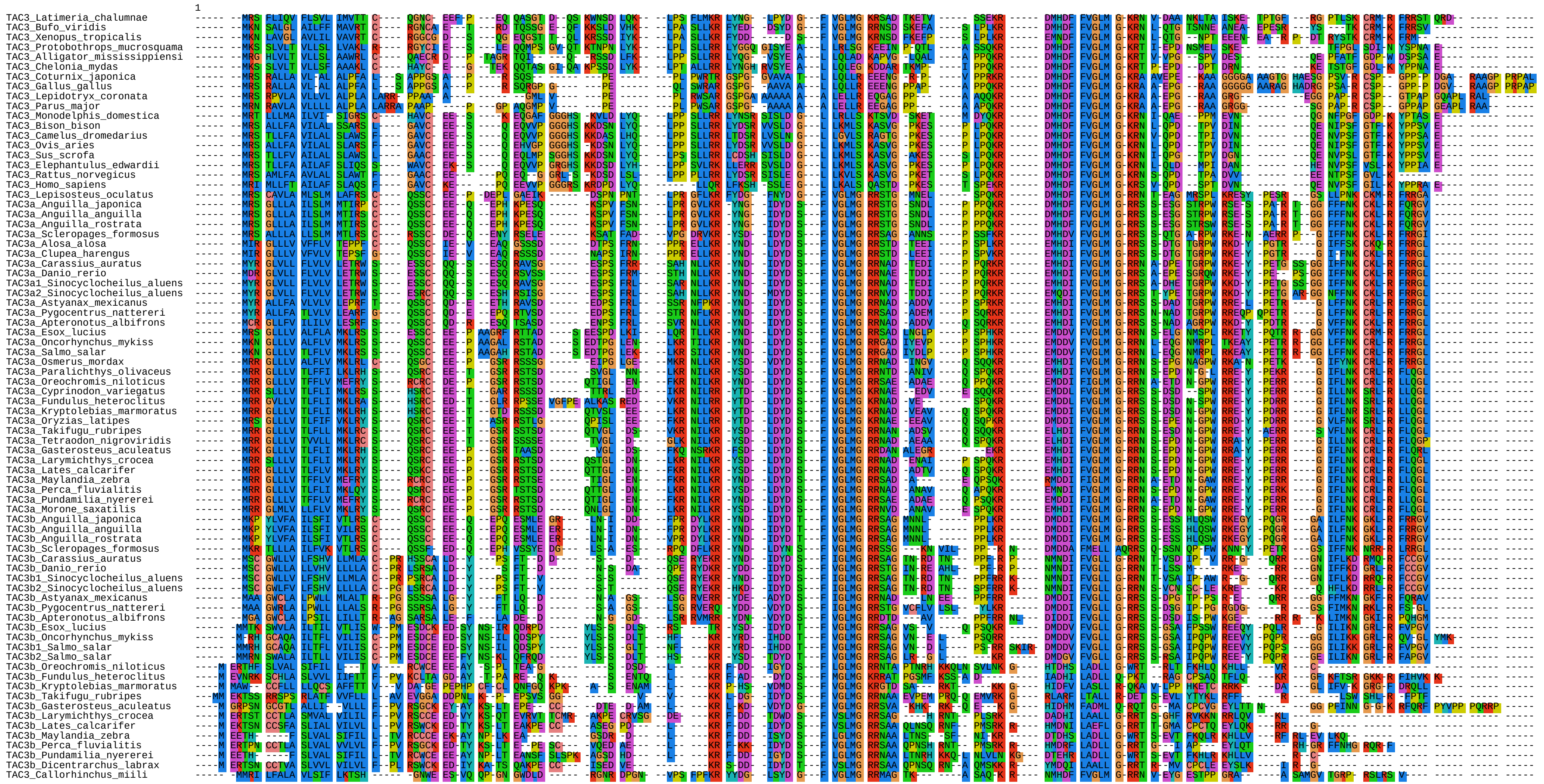
1
TAC3_Latimeria_chalumnae
TAC3_Bufo_viridis
TAC3_Xenopus_tropicalis
TAC3_Protobothrops_mucrosquama
TAC3_Alligator_mississippiensi
TAC3_Chelonia_mydas
TAC3_Coturnix_japonica
TAC3_Gallus_gallus
TAC3_Lepidotryx_coronata
TAC3_Parus_major
TAC3_Monodelphis_domestica
TAC3_Bison_bison
TAC3_Camelus_dromedarius
TAC3_Ovis_aries
TAC3_Sus_scrofa
TAC3_Elephantulus_edwardii
TAC3_Rattus_norvegicus
TAC3_Homo_sapiens
TAC3_Lepisosteus_oculatus
TAC3a_Anguilla_japonica
TAC3a_Anguilla_anguilla
TAC3a_Anguilla_rostrata
TAC3a_Scleropages_formosus
TAC3a_Alosa_alosa
TAC3a_Clupea_harengus
TAC3a_Carassius_auratus
TAC3a_Danio_rerio
TAC3a1_Sinocyclocheilus_aluens
TAC3a2_Sinocyclocheilus_aluens
TAC3a_Astyanax_mexicanus
TAC3a_Pygocentrus_nattereri
TAC3a_Apteronotus_albifrons
TAC3a_Esox_lucius
TAC3a_Oncorhynchus_mykiss
TAC3a_Salmo_salar
TAC3a_Osmerus_mordax
TAC3a_Paralichthys_olivaceus
TAC3a_Oreochromis_niloticus
TAC3a_Cyprinodon_variegatus
TAC3a_Fundulus_heteroclitus
TAC3a_Kryptolebias_marmoratus
TAC3a_Oryzias_latipes
TAC3a_Takifugu_rubripes
TAC3a_Tetraodon_nigroviridis
TAC3a_Gasterosteus_aculeatus
TAC3a_Larymichthys_crocea
TAC3a_Lates_calcarifer
TAC3a_Maylandia_zebra
TAC3a_Perca_fluvialitis
TAC3a_Pundamilia_nyererei
TAC3a_Morone_saxatilis
TAC3b_Anguilla_japonica
TAC3b_Anguilla_anguilla
TAC3b_Anguilla_rostrata
TAC3b_Scleropages_formosus
TAC3b_Carassius_auratus
TAC3b_Danio_rerio
TAC3b1_Sinocyclocheilus_aluens
TAC3b2_Sinocyclocheilus_aluens
TAC3b_Astyanax_mexicanus
TAC3b_Pygocentrus_nattereri
TAC3b_Apteronotus_albifrons
TAC3b_Esox_lucius
TAC3b_Oncorhynchus_mykiss
TAC3b1_Salmo_salar
TAC3b2_Salmo_salar
TAC3b_Oreochromis_niloticus
TAC3b_Fundulus_heteroclitus
TAC3b_Kryptolebias_marmoratus
TAC3b_Takifugu_rubripes
TAC3b_Gasterosteus_aculeatus
TAC3b_Larymichthys_crocea
TAC3b_Lates_calcarifer
TAC3b_Maylandia_zebra
TAC3b_Perca_fluvialitis
TAC3b_Pundamilia_nyererei
TAC3b_Dicentrarchus_labrax
TAC3_Callorhinchus_miili

**Supplementary Figure 2.** Alignment of 28 C1GALT1 amino-acid sequences used in the phylogeny analysis (Fig S3). The amino-acid residues with similar physico-chemical properties are in the same color.

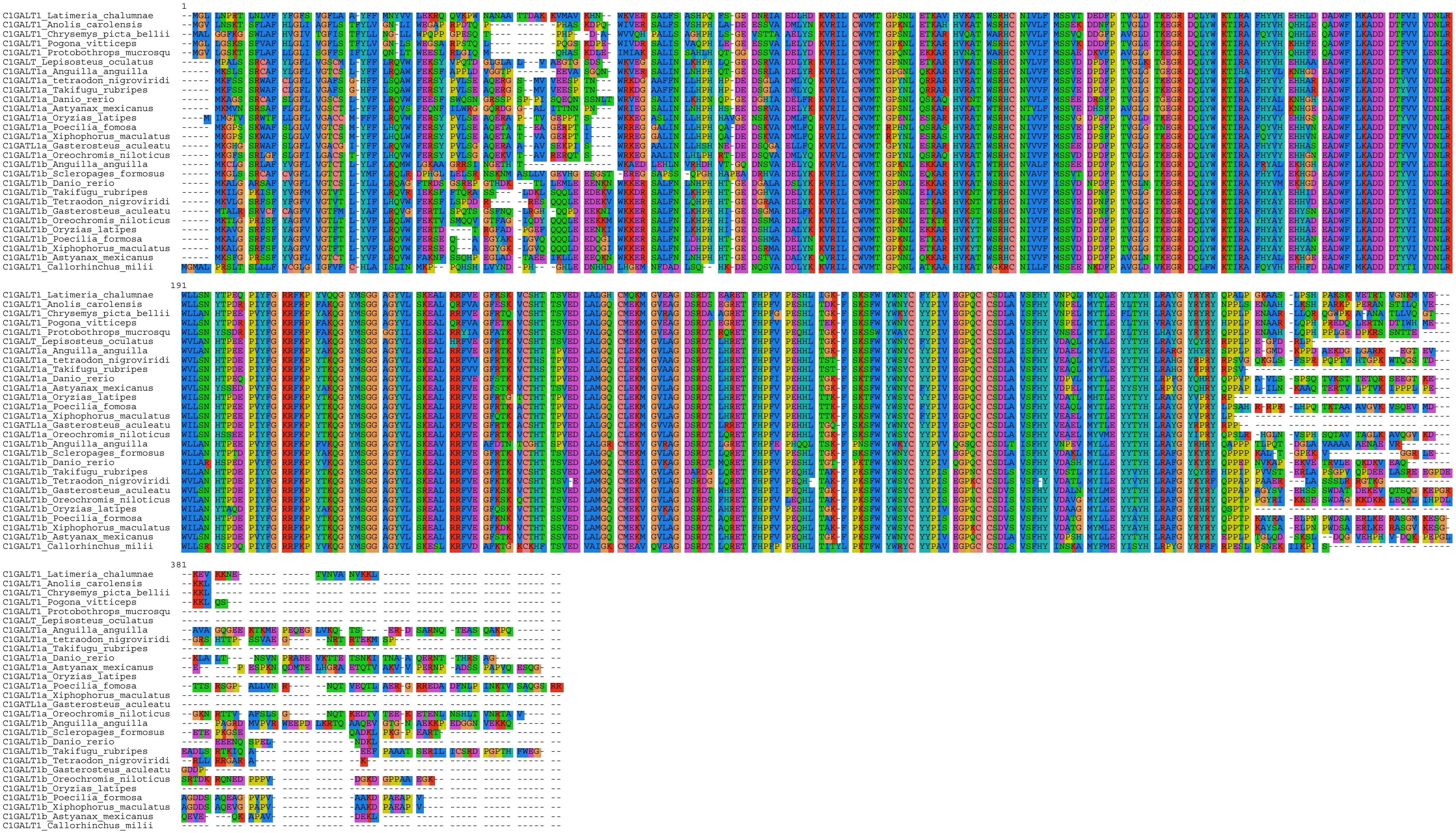
1
191
381
C1GALT1_Latimeria_chalumnae
C1GALT1_Anolis_carolensis
C1GALT1_Chrysemys_picta_bellii
C1GALT1_Pogona_vitticeps
C1GALT1_Protobothrops_mucrosqu
C1GALT_Lepisosteus_oculatus
C1GALT1a_Anguilla_anguilla
C1GALT1a_tetraodon_nigroviridi
C1GALT1a_Takifugu_rubripes
C1GALT1a_Danio_rerio
C1GALT1a_Astyanax_mexicanus
C1GALT1a_Oryzias_latipes
C1GALT1a_Poecilia_fomosa
C1GALT1a_Xiphophorus_maculatus
C1GATL1a_Gasterosteus_aculeatu
C1GALT1a_Oreochromis_niloticus
C1GALT1b_Anguilla_anguilla
C1GALT1b_Scleropages_formosus
C1GALT1b_Danio_rerio
C1GALT1b_Takifugu_rubripes
C1GALT1b_Tetraodon_nigroviridi
C1GALT1b_Gasterosteus_aculeatu
C1GALT1b_Oreochromis_niloticus
C1GALT1b_Oryzias_latipes
C1GALT1b_Poecilia_formosa
C1GALT1b_Xiphophorus_maculatus
C1GALT1b_Astyanax_mexicanus
C1GALT1_Callorhinchus_milii

**Supplementary Figure 3.** Consensus phylogenetic tree of vertebrate C1GALT1. This phylogenetic tree was based on the amino-acid sequences of C1GALT1 (Table S4) using the Maximum Likelihood method with 1,000 bootstrap replicates. The number shown at each branch node indicates the bootstrap value (%); only values above 50% are indicated. The tree was rooted using the C1GALT1 sequence of the elephant shark *Callorhinchus milii*. The sequences corresponding to paralogon "a" and "b" are framed in blue and red, respectively.

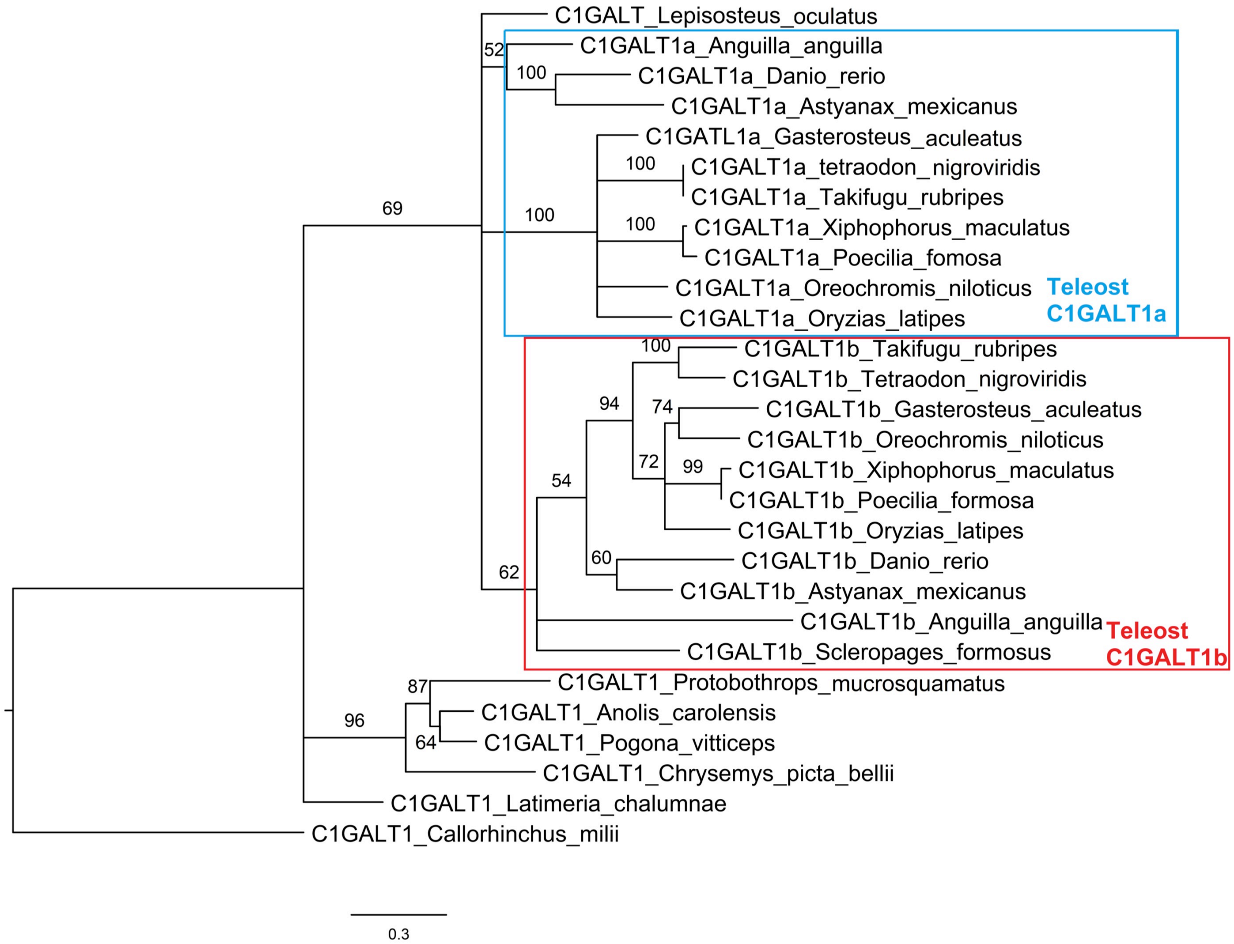


**Supplementary Figure 4.** Alignment of 30 B4GALTN1 amino-acid sequences used in the phylogenetic (Fig S5). The amino-acid residues with similar physico-chemical are in the same color.

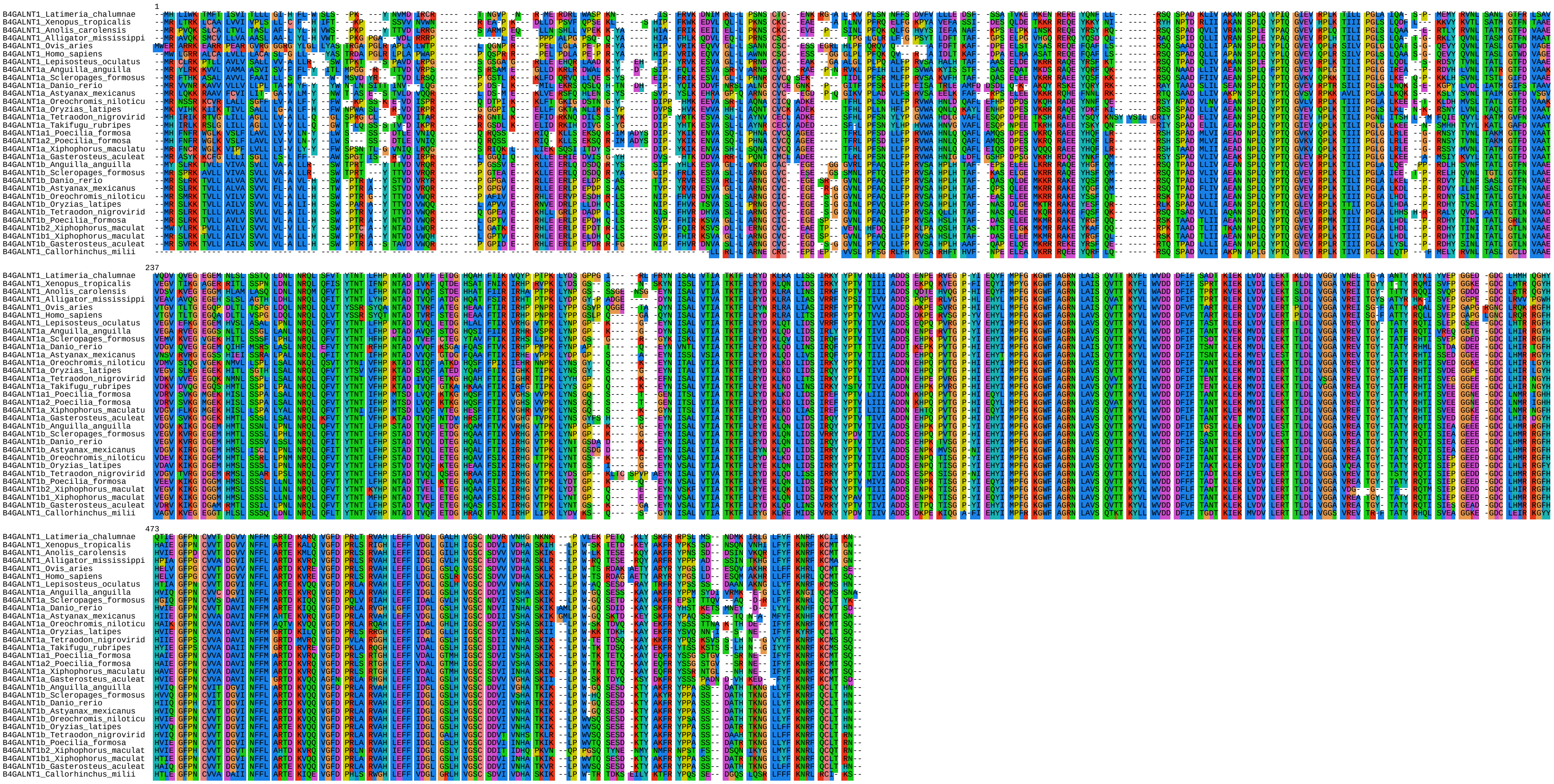
1
237
473
B4GALNT1_Latimeria_chalumnae
B4GALNT1_Xenopus_tropicalis
B4GALNT1_Anolis_carolensis
B4GALNT1_Alligator_mississippi
B4GALNT1_Ovis_aries
B4GALNT1_Homo_sapiens
B4GALNT1_Lepisosteus_oculatus
B4GALNT1a_Anguilla_anguilla
B4GALNT1a_Scleropages_formosus
B4GALNT1a_Danio_rerio
B4GALNT1a_Astyanax_mexicanus
B4GALNT1a_Oreochromis_niloticu
B4GALNT1a_Oryzias_latipes
B4GALNT1a_Tetraodon_nigrovirid
B4GALNT1a_Takifugu_rubripes
B4GALNT1a1_Poecilia_formosa
B4GALNT1a2_Poecilia_formosa
B4GALNT1a_Xiphophorus_maculatu
B4GALNT1a_Gasterosteus_aculeat
B4GALNT1b_Anguilla_anguilla
B4GALNT1b_Scleropages_formosus
B4GALNT1b_Danio_rerio
B4GALNT1b_Astyanax_mexicanus
B4GALNT1b_Oreochromis_niloticu
B4GALNT1b_Oryzias_latipes
B4GALNT1b_Tetraodon_nigrovirid
B4GALNT1b_Poecilia_formosa
B4GALNT1b2_Xiphophorus_maculat
B4GALNT1b1_Xiphophorus_maculat
B4GALNT1b_Gasterosteus_aculeat
B4GALNT1_Callorhinchus_milii

**Supplementary Figure 5.** Consensus phylogenetic tree of vertebrate B4GALNT1. This phylogenetic tree was based on the amino-acid sequences of B4GALNT1 (Table S5) using the Maximum Likelihood method with 1,000 bootstrap replicates. The number shown at each branch node indicates the bootstrap value (%); only values above 40% are indicated. The tree was rooted using the B4GALNT1 sequence of the elephant shark *Callorhinchus milii*. The sequences corresponding to paralogon “a” and “b” are framed in blue and red, respectively.

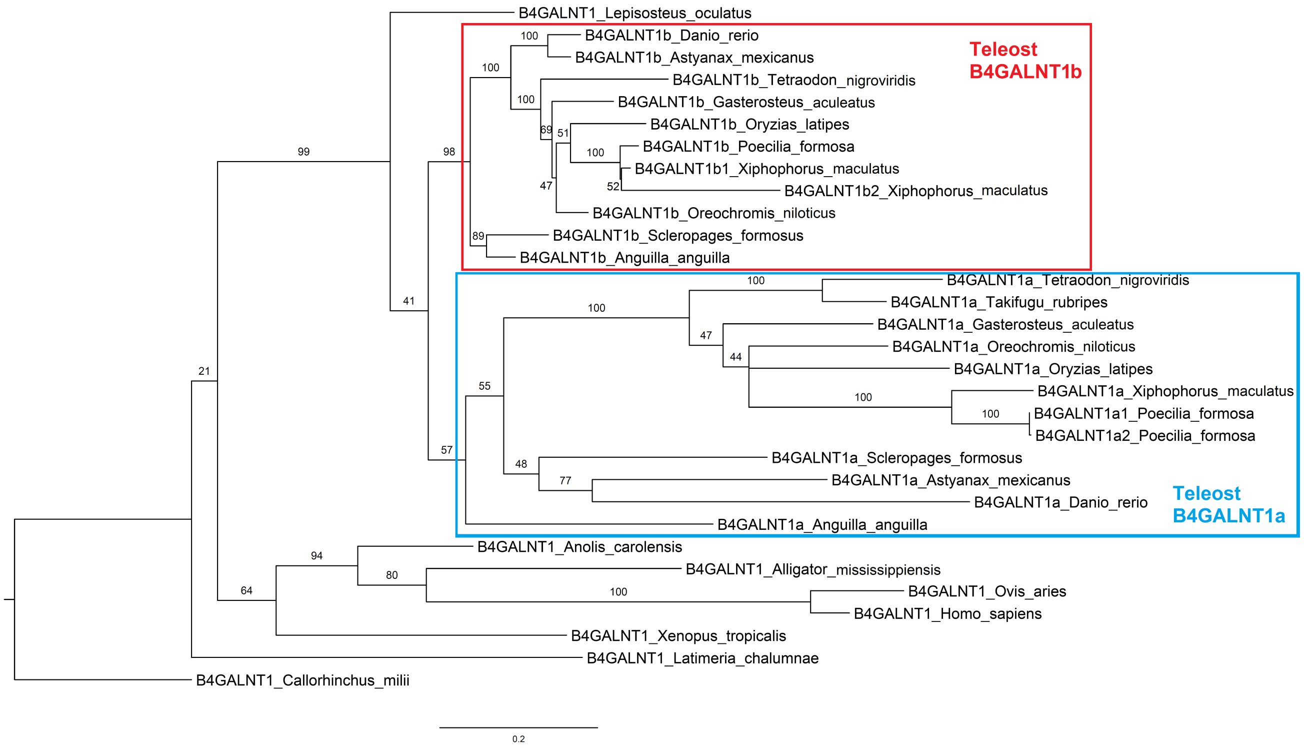


## 2. Supplementary Tables

**Supplementary Table 1.** Name and NCBI accession number of the TAC3 pre-pro-peptides used in the alignment (Fig S1) and phylogeny analysis (Fig2).

| Pre-pro-peptide | Species | Accession number | Annotation provider |
|---|---|---|---|
| *TAC3* | *Latimeria chalumnae* | *XM_005986188.1* | *NCBI* |
| *TAC3* | *Bufo viridis* | *predicted* | *Present article* |
| *TAC3* | *Xenopus tropicalis* | *NM_001267891.2* | *Biran et al. 2012 (ref 5)* |
| *TAC3* | *Protobothrops mucrosquamatus* | *XM_015822461.1* | *NCBI* |
| *TAC3* | *Alligator mississippiensis* | *BK008115.1* | *Biran et al. 2012 (ref 5)* |
| *TAC3* | *Chelonia mydas* | *XM_007066589.1* | *NCBI* |
| *TAC3* | *Coturnix japonica* | *XM_015887066.1* | *NCBI* |

| | | | |
|---|---|---|---|
| *TAC3* | *Gallus gallus* | *XM_015300406.1* | *NCBI* |
| *TAC3* | *Lepidothryx coronata* | *XM_017836603.1* | *NCBI* |
| *TAC3* | *Parus major* | *XM_015615656.1* | *NCBI* |
| *TAC3* | *Monodelphis domestica* | *XM_007506244.2* | *NCBI* |
| *TAC3* | *Bison bison* | *XM_010839305.1* | *NCBI* |
| *TAC3* | *Camelus dromedarius* | *XM_010990978.1* | *NCBI* |
| *TAC3* | *Ovis aries* | *XM_015094789.1* | *NCBI* |
| *TAC3* | *Sus scrofa* | *NM_001007196.1* | *Li et al. 2005* |
| *TAC3* | *Elephantulus edwardii* | *XM_006897674.1* | *NCBI* |
| *TAC3* | *Rattus norvegicus* | *NM_019162.2* | *Ruiz Pino et al. 2015* |
| *TAC3* | *Homo sapiens* | *KJ892241.1* | *Yang et al. 2011* |
| *TAC3* | *Lepisosteus oculatus* | *XM_015344463.1* | *NCBI* |
| *TAC3a* | *Anguilla anguilla* | *clonned* | *Present article* |
| *TAC3b* | *Anguilla anguilla* | *clonned* | *Present article* |
| *TAC3a* | *Anguilla japonica* | *predicted* | *Present article* |
| *TAC3b* | *Anguilla japonica* | *predicted* | *Present article* |
| *TAC3a* | *Anguilla rostrata* | *predicted* | *Present article* |
| *TAC3b* | *Anguilla rostrata* | *predicted* | *Present article* |
| *TAC3a* | *Scleropages formosus* | *predicted* | *Present article* |
| *TAC3b* | *Scleropages formosus* | *predicted* | *Present article* |
| *TAC3a* | *Alosa alosa* | *predicted* | *Present article* |
| *TAC3a* | *Clupea harengus* | *XM_012824242.1* | *NCBI* |
| *TAC3a* | *Carassius auratus* | *KF177342.1* | *Qi et al. 2015 (ref 32)* |
| *TAC3b* | *Carassius auratus* | *KF177343.1* | *Qi et al. 2015 (ref 32)* |
| *TAC3a* | *Danio rerio* | *JN392856.1* | *Biran et al. 2012 (ref 5)* |
| *TAC3b* | *Danio rerio* | *JN392857.1* | *Biran et al. 2012 (ref 5)* |
| *TAC3a1* | *Sinocyclocheilus anshuiensis* | *XM_016478326.1* | *NCBI* |
| *TAC3a2* | *Sinocyclocheilus anshuiensis* | *XM_016495847.1* | *NCBI* |
| *TAC3b1* | *Sinocyclocheilus anshuiensis* | *predicted* | *Present article* |
| *TAC3b2* | *Sinocyclocheilus anshuiensis* | *predicted* | *Present article* |
| *TAC3a* | *Astianax mexicanus* | *XM_007246760.3* | *NCBI* |
| *TAC3b* | *Astianax mexicanus* | *predicted* | *Present article* |
| *TAC3a* | *Pygocentrus nattereri* | *XM_017706258.1* | *NCBI* |
| *TAC3b* | *Pygocentrus nattereri* | *predicted* | *Present article* |
| *TAC3a* | *Apteronotus albifrons* | *predicted* | *Present article* |
| *TAC3b* | *Apteronotus albifrons* | *predicted* | *Present article* |
| *TAC3a* | *Esox lucius* | *XM_010875105.3* | *NCBI* |
| *TAC3b* | *Esox lucius* | *XM_010881641.2* | *NCBI* |
| *TAC3a* | *Oncorhynchus mykiss* | *XM_021565858.1* | *NCBI* |
| *TAC3b* | *Oncorhynchus mykiss* | *predicted* | *Present article* |
| *TAC3a* | *Salmo salar* | *BK008102.1* | *Biran et al. 2012 (ref 5)* |
| *TAC3b1* | *Salmo salar* | *BK008103.1* | *Biran et al. 2012 (ref 5)* |
| *TAC3b2* | *Salmo salar* | *predicted* | *Present article* |

| | | | |
|---|---|---|---|
| *TAC3a* | *Osmerus mordax* | *BK008111.1* | *Biran et al. 2012 (ref 5)* |
| *TAC3a* | *Paralichthys olivaceus* | *XM_020089538.1* | *NCBI* |
| *TAC3a* | *Oreochromis niloticus* | *KF471673.1* | *Biran et al. 2014 (ref 31)* |
| *TAC3b* | *Oreochromis niloticus* | *predicted* | *Present article* |
| *TAC3a* | *Cyprinodon variegatus* | *XM_015377892.1* | *NCBI* |
| *TAC3a* | *Fundulus heteroclitus* | *predicted* | *Present article* |
| *TAC3b* | *Fundulus heteroclitus* | *predicted* | *Present article* |
| *TAC3a* | *Kryptolebias marmoratus* | *XM_017430900.1* | *NCBI* |
| *TAC3b* | *Kryptolebias marmoratus* | *predicted* | *Present article* |
| *TAC3a* | *Oryzas latipes* | *NM_001278903.1* | *Biran et al. 2012 (ref 5)* |
| *TAC3a* | *Takifugu rubripes* | *XM_011621888.1* | *NCBI* |
| *TAC3b* | *Takifugu rubripes* | *predicted* | *Present article* |
| *TAC3a* | *Tetraodon nigroviridis* | *CR713079* | *Jaillon et al. 2004* |
| *TAC3a* | *Gasterosteus acculeatus* | *predicted* | *Present article* |
| *TAC3b* | *Gasterosteus acculeatus* | *predicted* | *Present article* |
| *TAC3a* | *Larymichthys crocea* | *XM_010747871.2* | *NCBI* |
| *TAC3b* | *Larymichthys crocea* | *predicted* | *Present article* |
| *TAC3a* | *Lates calcarifer* | *XM_018666039.1* | *NCBI* |
| *TAC3b* | *Lates calcarifer* | *XR_001960617.1* | *NCBI* |
| *TAC3a* | *Maylandia zebra* | *predicted* | *Present article* |
| *TAC3b* | *Maylandia zebra* | *predicted* | *Present article* |
| *TAC3a* | *Perca fluvialitis* | *predicted* | *Present article* |
| *TAC3b* | *Perca fluvialitis* | *predicted* | *Present article* |
| *TAC3a* | *Pundamilia nyererei* | *XM_005732601.1* | *NCBI* |
| *TAC3b* | *Pundamilia nyererei* | *predicted* | *Present article* |
| *TAC3b* | *Dicentrarchus labrax* | *BK008116.1* | *Biran et al. 2012 (ref 5)* |
| *TAC3a* | *Morone saxatilis* | *KT361626.1* | *Zmora et al. 2017 (ref 34)* |
| *TAC3* | *Callorhinchus milii* | *XM_007885087.1* | *NCBI* |


Li SJ et al. Somatic cell hybrid and RH mapping of the *tachykinin 3* gene to porcine chromosome 5. *Animal genetics* (2005) 36:90-91.

Ruiz Pino F et al. Effects and interactions of tachykinins and dynorphin on FSH and LH secretion in developing adult rats. *Endocrinology* (2015) 156:576-588.

Yang X et al. A public genome-scale lentiviral expression library of human ORFs. *Nature Methods* (2011) 8:659-661.

Jaillon O et al. Genome duplication in the teleost fish *Tetraodon nigroviridis* reveals the early vertebrate proto-karyotype. *Nature* (2004) 431:946-957.

**Supplementary Table 2.** List of PCR and qPCR primers. Gene names are indicated by the same acronym used in the text.

| Primer ID | PCR type | 5' - 3' Sequence | Tm (C ) | Amplicon size (bp) | References |
|---|---|---|---|---|---|
| TAC3a F | qPCR | *ATCCGATCATGCCAGTCCAG* | 59.4 | 140 | Present article |
| TAC3a R | qPCR | *ATAAGGCCCACGAAGCTGTC* | 59.4 | | |
| TAC3b F | qPCR | *CATTGACAACTTCCCGCGTG* | 59.4 | 190 | Present article |
| TAC3b R | qPCR | *TCTCCAGGATTGCAGGTGAC* | 59.4 | | |
| LHβ F | qPCR | *TCACCTCCTTGTTTCTGCTG* | 57.43 | 149 | Aroua et al. 2007 (ref 53) |
| LHβ R | qPCR | *TAGCTTGGGTCCTTGGTGATG* | 60.83 | | |
| FSHβ F | qPCR | *TCTCGCCAACATCTCCATC* | 58.09 | 100 | Aroua et al. 2007 (ref 53) |
| FSHβ R | qPCR | *AGAATCCTGGGTGAAGCACA* | 59.09 | | |
| GnRH-R2 F | qPCR | *TCACCTTCTCCTGCCTCTTC* | 59.4 | 105 | Peñaranda et al. 2014 (ref 55) |
| GnRH-R2 R | qPCR | *TTGGAAGATGCCTTCCCTTT* | 55.3 | | |
| β-actin F | qPCR | *AGTATTTGCGCTCGGGTG* | 58.25 | 226 | Aroua et al. 2007 (ref 53) |
| β-actin R | qPCR | *CAGCCTTCCTTCCTGGGT* | 58.6 | | |
| GPα F | qPCR | *TGCCGACTCCAGGAGAATAA* | 59.21 | 184 | Aroua et al. 2007 (ref 53) |
| GPα R | qPCR | *TGTTATCCAGCCTTGTCACC* | 57.01 | | |
| TSHβ F | qPCR | *GCTTCTGCTATTCCCTGGAC* | 59.4 | 295 | Maugars et al. 2014 (ref 54) |
| TSHβ R | qPCR | *AGGGTCTGGATGTAGTTGCTC* | 59.8 | | |
| GH F | qPCR | *ACCGTCACCTACATCCTTCAT* | 57.9 | 183 | Aroua et al. 2007 (ref 53) |
| GH R | qPCR | *AAATCGGATGGGTACTTGCTG* | 57.9 | | |
| TAC3a UTR F | PCR | *AACTCTCCGCTTCCACATCA* | 57.3 | 559 | Present article |
| TAC3a UTR R | PCR | *CTGGAACCAAAAGAAGGAGCA* | 57.9 | | |
| TAC3b UTR F | PCR | *AACTCAACGGCTGACAGAGA* | 57.3 | 429 | Present article |
| TAC3b UTR R | PCR | *CCCAAGTTTCACGGTTTCCA* | 57.3 | | |
| TAC3aNES F | PCR | *TCTCCGCTTCCACATCAGAG* | 59.4 | 330 | Present article |
| TAC3aNES R | PCR | *AGAAGGAGCAAACTAGGCAG* | 57.3 | | |
| TAC3bNES F | PCR | *CGGCTGACAGAGAGACACAA* | 59.4 | 521 | Present article |
| TAC3bNES R | PCR | *CATTCCAGTAAAGGGCGAAAAG* | 58.4 | | |
| TAC3aORF F | PCR | *GGCGATCCTGTCCCTTATGA* | 59.4 | 508 | Present article |
| TAC3aORF R | PCR | *CTACACGCCGCGCTGAAA* | 58.2 | | |
| TAC3bORF F | PCR | *TACTTGGTGTTCGCGATCCT* | 57.3 | 320 | Present article |
| TAC3bORF R | PCR | *TTAAACACCGCGGCGAAAC* | 56.7 | | |

**Supplementary Table 3.** Start and end positions of genes used for the synteny analysis (Fig 3). Species are separated as follows: Spotted gar (A), European eel (B), Golden arowana (C), Atlantic herring (D), Zebrafish (E), Medaka (F), Tilapia (G), Stickleback (H), Fugu (I).

A- **Spotted gar**

| Gene name | Chromosome | Strand | Start | End | Name |
|---|---|---|---|---|---|
| *APOF* | *4* | *-* | *18360102* | *18360599* | *predicted* |
| *b4galnt1* | *4* | *+* | *18,451,123* | *18,469,616* | *ENSLOCG00000007549* |
| *c1galt1* | *4* | *-* | *18,413,115* | *18,417,342* | *ENSLOCG00000007520* |
| *os9* | *4* | *-* | *18,424,244* | *18,440,368* | *ENSLOCG00000007533* |
| *slc26a10* | *4* | *-* | *18,475,722* | *18,488,327* | *ENSLOCG00000007565* |
| *sp5l* | *4* | *+* | *18,530,676* | *18,538,905* | *ENSLOCG00000007577* |
| *stat2* | *4* | *-* | *18,320,440* | *18,348,846* | *ENSLOCG00000007484* |
| *TAC3* | *4* | *+* | *18,400,010* | *18,406,027* | *ENSLOCG00000007508* |

**B European eel**

| Gene name | Scaffold | Scaffold Illumina | Strand | Start | End | Name |
|---|---|---|---|---|---|---|
| *APOFa* | *NOT FOUND* | *NOT FOUND* | *NOT FOUND* | *NOT FOUND* | *NOT FOUND* | *NOT FOUND* |
| *APOFb* | *1064* | *7234 + 41297 + 393457 + 371373 + 215787* | *+* | *138304* | *141388* | *predicted* |
| *b4galnt1a* | *1298* | *926* | *+* | *305580* | *332147* | *DAA_B4GN1.4.4* |
| *b4galnt1b* | *1064* | *997* | *+* | *63122* | *43899* | *DAA_B4GN1.1.4* |
| *c1galt1a* | *1298* | *926* | *-* | *300490* | *294895* | *g8752* |
| *c1galt1b* | *1064* | *997* | *+* | *92158* | *100195* | *DAA_LOC101071001.1.1* |
| *os9a* | *NOT FOUND* | *NOT FOUND* | *NOT FOUND* | *NOT FOUND* | *NOT FOUND* | *NOT FOUND* |
| *os9b* | *1064* | *997* | *+* | *73850* | *91790* | *g9139* |
| *slc26a10a* | *1298* | *926* | *-* | *358535* | *336898* | *g8754* |
| *slc26a10b* | *NOT FOUND* | *NOT FOUND* | *NOT FOUND* | *NOT FOUND* | *NOT FOUND* | *NOT FOUND* |
| *sp5la* | *1298* | *279* | *+* | *374224* | *376850* | *g4163* |
| *sp5lb* | *1064* | *259 + 264931* | *-* | *21439* | *20372* | *predicted* |
| *stat2a* | *NOT FOUND* | *NOT FOUND* | *NOT FOUND* | *NOT FOUND* | *NOT FOUND* | *NOT FOUND* |
| *stat2b* | *1064* | *11124+contig1975* | *-* | *159207* | *144007* | *g25275* |
| *TAC3a* | *1298* | *926* | *+* | *285648* | *293166* | *predicted* |
| *TAC3b* | *1064* | *997* | *-* | *116556* | *102959* | *predicted* |

**B- Golden arowana**

| Gene name | Scaffold | Strand | Start | End | Name |
|---|---|---|---|---|---|
| *APOFa* | *NOT FOUND* | *NOT FOUND* | *NOT FOUND* | *NOT FOUND* | *NOT FOUND* |
| *APOFb* | *NW_017372270.1* | *-* | *6967277* | *6964631* | *XP_018581191.1* |
| *b4galnt1a* | *NW_017372100.1* | *+* | *10512021* | *10522571* | *XM_018760130.1* |
| *b4galnt1b* | *NW_017372270.1* | *+* | *7007769* | *7028122* | *XM_018726028.1* |
| *c1galt1a* | *NOT FOUND* | *NOT FOUND* | *NOT FOUND* | *NOT FOUND* | *NOT FOUND* |
| *c1galt1b* | *NW_017372270.1* | *-* | *6983706* | *6995592* | *XP_018580957.1* |
| *os9a* | *NOT FOUND* | *NOT FOUND* | *NOT FOUND* | *NOT FOUND* | *NOT FOUND* |
| *os9b* | *NW_017372270.1* | *+* | *6995931* | *7005853* | *XP_018580723.1* |
| *slc26a10a* | *NW_017372100.1* | *-* | *10524752* | *10534501* | *XP_018615803.1* |
| *slc26a10b* | *NOT FOUND* | *NOT FOUND* | *NOT FOUND* | *NOT FOUND* | *NOT FOUND* |
| *sp5la* | *NW_017372100.1* | *+* | *10562179* | *10562428* | *XP_01861590.1* |
| *sp5lb* | *NW_017372270.1* | *+* | *7046475* | *7049846* | *XP_018580928.1* |
| *stat2a* | *NOT FOUND* | *NOT FOUND* | *NOT FOUND* | *NOT FOUND* | *NOT FOUND* |
| *stat2b* | *NW_017372270.1* | *-* | *6944862* | *6962876* | *XP_018581189.1* |
| *TAC3a* | *NW_017372100.1* | *+* | *10486419* | *10490938* | *predicted* |
| *TAC3b* | *NW_017372270.1* | *+* | *6983657* | *6988623* | *predicted* |

## C- Atlantic herring

| Gene name | Scaffold | Strand | Start | End | Name |
|---|---|---|---|---|---|
| *APOFa* | *NOT FOUND* | *NOT FOUND* | *NOT FOUND* | *NOT FOUND* | *NOT FOUND* |
| *APOFb* | *NW_012220764* | *+* | *1247562* | *1251616* | *XM_012824856.1* |
| *b4galnt1a* | *NW_012220693* | *+* | *993981* | *1036038* | *XM_012824238.1* |
| *b4galnt1b* | *NOT FOUND* | *NOT FOUND* | *NOT FOUND* | *NOT FOUND* | *NOT FOUND* |
| *c1galt1a* | *NW_012220693* | *-* | *983589* | *976247* | *XM_012824193.1* |
| *c1galt1b* | *NW_012220764* | *+* | *1242824* | *1245850* | *XM_012824880.1* |
| *os9a* | *NOT FOUND* | *NOT FOUND* | *NOT FOUND* | *NOT FOUND* | *NOT FOUND* |
| *os9b* | *NW_012220764* | *+* | *1540408* | *1572781* | *XM_012824950.1* |
| *slc26a10a* | *NW_012220693* | *-* | *1049705* | *1043574* | *XM_012824197.1* |
| *slc26a10b* | *NOT FOUND* | *NOT FOUND* | *NOT FOUND* | *NOT FOUND* | *NOT FOUND* |
| *sp5la* | *NW_012220693* | | *1076150* | *1079368* | *XM_012824198.1* |
| *sp5lb* | *NOT FOUND* | *NOT FOUND* | *NOT FOUND* | *NOT FOUND* | *NOT FOUND* |
| *stat2a* | *NOT FOUND* | *NOT FOUND* | *NOT FOUND* | *NOT FOUND* | *NOT FOUND* |
| *stat2b* | *NW_012220764* | *+* | *1256363* | *1271064* | *XM_012824856.1* |
| *TAC3a* | *NW_012220693* | *+* | *971516* | *972842* | *XM_012824242.1* |
| *TAC3b* | *NOT FOUND* | *NOT FOUND* | *NOT FOUND* | *NOT FOUND* | *NOT FOUND* |

**D- Zebrafish**

| Gene name | Scaffold | Strand | Start | End | Name |
|---|---|---|---|---|---|
| *APOFa* | *NOT FOUND* | *NOT FOUND* | *NOT FOUND* | *NOT FOUND* | *NOT FOUND* |
| *APOFb* | *6* | *-* | *39,164,758* | *39,170,196* | *ENSDARG00000090980* |
| *b4galnt1a* | *23* | *-* | *28,169,082* | *28,197,226* | *ENSDARG00000061520* |
| *b4galnt1b* | *6* | *+* | *39,225,062* | *39,255,315* | *ENSDARG00000077352* |
| *c1galt1a* | *23* | *+* | *28,165,542* | *28,212,580* | *ENSDARG00000055585* |
| *c1galt1b* | *6* | *-* | *39,192,866* | *39,201,534* | *ENSDARG00000055561* |
| *os9a* | *NOT FOUND* | *NOT FOUND* | *NOT FOUND* | *NOT FOUND* | *NOT FOUND* |
| *os9b* | *6* | *-* | *39,205,149* | *39,221,073* | *ENSDARG00000020301* |
| *slc26a10a* | *23* | *+* | *28,151,412* | *28,164,568* | *ENSDARG00000078800* |
| *slc26a10b* | *NOT FOUND* | *NOT FOUND* | *NOT FOUND* | *NOT FOUND* | *NOT FOUND* |
| *sp5la* | *23* | *-* | *28,096,555* | *28,099,402* | *ENSDARG00000010124* |
| *sp5lb* | *NOT FOUND* | *NOT FOUND* | *NOT FOUND* | *NOT FOUND* | *NOT FOUND* |
| *stat2a* | *NOT FOUND* | *NOT FOUND* | *NOT FOUND* | *NOT FOUND* | *NOT FOUND* |
| *stat2b* | *6* | *-* | *39,126,008* | *39,162,886* | *ENSDARG00000031647* |
| *TAC3a* | *23* | *-* | *28,212,948* | *28,214,878* | *ENSDARG00000093089* |
| *TAC3b* | *6* | *+* | *39,186,700* | *39,190,121* | *ENSDARG00000097596* |

## E- Medaka

| Gene name | Scaffold | Strand | Start | End | Name |
|---|---|---|---|---|---|
| *APOFa* | *NOT FOUND* | *NOT FOUND* | *NOT FOUND* | *NOT FOUND* | *NOT FOUND* |
| *APOFb* | *5* | *+* | *2,465,799* | *2,468,618* | *ENSORLG00000001646* |
| *b4galnt1a* | *7* | *+* | *12,271,869* | *12,282,550* | *XM_020704642.1* |
| *b4galnt1b* | *5* | *-* | *2,411,270* | *2,421,401* | *ENSORLG00000001618* |
| *c1galt1a* | *7* | *-* | *12,259,498* | *12,263,422* | *ENSORLG00000007498* |
| *c1galt1b* | *5* | *+* | *2,454,606* | *2,459,872* | *ENSORLG00000001643* |
| *os9a* | *NOT FOUND* | *NOT FOUND* | *NOT FOUND* | *NOT FOUND* | *NOT FOUND* |
| *os9b* | *5* | *+* | *2,437,952* | *2,449,467* | *ENSORLG00000001640* |
| *slc26a10a* | *7* | *-* | *12,286,683* | *12,291,323* | *ENSORLG00000007519* |
| *slc26a10b* | *NOT FOUND* | *NOT FOUND* | *NOT FOUND* | *NOT FOUND* | *NOT FOUND* |
| *sp5la* | *7* | *+* | *12,315,360* | *12,317,000* | *ENSORLG00000007526* |
| *sp5lb* | *NOT FOUND* | *NOT FOUND* | *NOT FOUND* | *NOT FOUND* | *NOT FOUND* |
| *stat2a* | *NOT FOUND* | *NOT FOUND* | *NOT FOUND* | *NOT FOUND* | *NOT FOUND* |
| *stat2b* | *5* | *+* | *2,471,673* | *2,476,751* | *ENSORLG00000001651* |
| *TAC3a* | *7* | *+* | *12,256,959* | *12,258,340* | *ENSORLG00000007492* |
| *TAC3b* | *NOT FOUND* | *NOT FOUND* | *NOT FOUND* | *NOT FOUND* | *NOT FOUND* |

## F- Tilapia

| Gene name | Scaffold | Strand | Start | End | Name |
|---|---|---|---|---|---|
| *APOFa* | *NOT FOUND* | *NOT FOUND* | *NOT FOUND* | *NOT FOUND* | *NOT FOUND* |
| *APOFb* | *GL831146.1* | *+* | *2,661,668* | *2,662,945* | *ENSONIG00000021171* |
| *b4galnt1a* | *GL831196.1* | *+* | *1,152,564* | *1,166,785* | *ENSONIG00000012109* |
| *b4galnt1b* | *GL831146.1* | *-* | *2,544,566* | *2,553,108* | *ENSONIG00000019085* |
| *c1galt1a* | *GL831196.1* | *-* | *1,125,478* | *1,130,612* | *ENSONIG00000012108* |
| *c1galt1b* | *GL831146.1* | *+* | *2,638,855* | *2,643,692* | *ENSONIG00000019092* |
| *os9a* | *NOT FOUND* | *NOT FOUND* | *NOT FOUND* | *NOT FOUND* | *NOT FOUND* |
| *os9b* | *GL831146.1* | *+* | *2,557,277* | *2,568,899* | *ENSONIG00000019088* |
| *slc26a10a* | *GL831190.1* | *-* | *1,171,402* | *1,178,570* | *ENSONIG00000012113* |
| *slc26a10b* | *NOT FOUND* | *NOT FOUND* | *NOT FOUND* | *NOT FOUND* | *NOT FOUND* |
| *sp5la* | *GL831196.1* | *+* | *1,197,578* | *1,200,437* | *ENSONIG00000012117* |
| *sp5lb* | *NOT FOUND* | *NOT FOUND* | *NOT FOUND* | *NOT FOUND* | *NOT FOUND* |
| *stat2a* | *NOT FOUND* | *NOT FOUND* | *NOT FOUND* | *NOT FOUND* | *NOT FOUND* |
| *stat2b* | *GL831146.1* | *+* | *2,668,069* | *2,677,496* | *ENSONIG00000019094* |
| *TAC3a* | *GL831196.1* | *-* | *1,123,378* | *1,124,183* | *NM_001311342.1* |
| *TAC3b* | *GL831146.1* | *-* | *2,647,978* | *2,653,087* | *predicted* |

## G- Stickleback

| Gene name | Scaffold | Strand | Start | End | Name |
|---|---|---|---|---|---|
| *APOFa* | *NOT FOUND* | *NOT FOUND* | *NOT FOUND* | *NOT FOUND* | *NOT FOUND* |
| *APOFb* | *27* | *-* | *1,921,736* | *1,923,064* | *ENSGACG00000000709* |
| *b4galnt1a* | *XII* | *+* | *11,114,611* | *11,120,117* | *Predicted* |
| *b4galnt1b* | *27* | *+* | *1,946,114* | *1,954,478* | *ENSGACG00000000713* |
| *c1galt1a* | *XII* | *-* | *11,106,920* | *11,109,223* | *ENSGACG00000008969* |
| *c1galt1b* | *27* | *+* | *1,930,982* | *1,935,286* | *ENSGACG00000000710* |
| *os9a* | *NOT FOUND* | *NOT FOUND* | *NOT FOUND* | *NOT FOUND* | *NOT FOUND* |
| *os9b* | *27* | *-* | *1,937,087* | *1,945,286* | *ENSGACG00000000711* |
| *slc26a10a* | *XII* | *-* | *11,123,251* | *11,128,216* | *ENSGACG00000008976* |
| *slc26a10b* | *NOT FOUND* | *NOT FOUND* | *NOT FOUND* | *NOT FOUND* | *NOT FOUND* |
| *sp5la* | *XII* | *+* | *11,144,529* | *11,145,968* | *ENSGACG00000008991* |
| *spl5b* | *NOT FOUND* | *NOT FOUND* | *NOT FOUND* | *NOT FOUND* | *NOT FOUND* |
| *stat2a* | *NOT FOUND* | *NOT FOUND* | *NOT FOUND* | *NOT FOUND* | *NOT FOUND* |
| *stat2b* | *27* | *-* | *1,909,074* | *1,917,435* | *ENSGACG00000000707* |
| *TAC3a* | *XII* | *+* | *11,104,847* | *11,105,875* | *Predicted* |
| *TAC3b* | *27* | *+* | *1,926,921* | *1,928,465* | *Predicted* |

## H- Fugu

| Gene name | Scaffold | Strand | Start | End | Name |
|---|---|---|---|---|---|
| *APOFa* | *NOT FOUND* | *NOT FOUND* | *NOT FOUND* | *NOT FOUND* | *NOT FOUND* |
| *APOFb* | *190* | *+* | *524,324* | *526,594* | *XM_003973211.2* |
| *b4galnt1a* | *66* | *-* | *585,666* | *592,675* | *Predicted* |
| *b4galnt1b* | *190* | *-* | *503,710* | *506,328* | *ENSTRUG00000012842* |
| *c1galt1a* | *66* | *+* | *595,206* | *597,481* | *ENSTRUG00000013143* |
| *c1galt1b* | *190* | *+* | *517,503* | *519,125* | *ENSTRUG00000013063* |
| *os9a* | *NOT FOUND* | *NOT FOUND* | *NOT FOUND* | *NOT FOUND* | *NOT FOUND* |
| *os9b* | *190* | *+* | *510,886* | *515,522* | *ENSTRUG00000012933* |
| *slc26a10a* | *66* | *+* | *579,350* | *583,361* | *ENSTRUG00000012857* |
| *slc26a10b* | *NOT FOUND* | *NOT FOUND* | *NOT FOUND* | *NOT FOUND* | *NOT FOUND* |
| *sp5la* | *66* | *-* | *0,564,330* | *565,723* | *ENSTRUG00000012835* |
| *sp5lb* | *NOT FOUND* | *NOT FOUND* | *NOT FOUND* | *NOT FOUND* | *NOT FOUND* |
| *stat2a* | *NOT FOUND* | *NOT FOUND* | *NOT FOUND* | *NOT FOUND* | *NOT FOUND* |
| *stat2b* | *190* | *+* | *528,290* | *532,939* | *ENSTRUG00000013090* |
| *TAC3a* | *66* | *-* | *598402* | *599852* | *predicted* |
| *TAC3b* | *190* | *+* | *521828* | *520608* | *predicted* |

**Supplementary Table 4.** Name and accession number of the C1GALT1 amino-acid sequences used in the alignment (Fig S2) and phylogeny analysis (Fig S3).

| Pre-pro-peptide | Species | Accession number | Annotation provider |
|---|---|---|---|
| *C1GALT1* | *Latimeria chalumnae* | *ENSLACG00000018257* | *Ensemble* |
| *C1GALT1* | *Anolis carolensis* | *ENSACAG00000006384* | *Ensemble* |
| *C1GALT1* | *Pogona vitticeps* | *XP_020633885.1* | *NCBI* |
| *C1GALT1* | *Protobothrops mucrosquamatus* | *XP_015672638.1* | *NCBI* |
| *C1GALT1* | *Chrysemys picta belli* | *XP_023956690.1* | *NCBI* |
| *C1GALT1* | *Lepisosteus oculatus* | *ENSLOCG00000007520* | *Ensemble* |
| *C1GALT1a* | *Anguilla anguilla* | *a_1298 DAA_C1GTA.2.3* | *Phylofish* |
| *C1GALT1b* | *Anguilla anguilla* | *b_1064 DAA_LOC101071001.1.1* | *Phylofish* |
| *C1GALT1b* | *Scleropages formosus* | *COB_C1GTB.1.1* | *Phylofish* |
| *C1GALT1a* | *Danio rerio* | *ENSDARG00000055585* | *Ensemble* |
| *C1GALT1b* | *Danio rerio* | *ENSDARG00000055561* | *Ensemble* |
| *C1GALT1a* | *Astyanax mexicanus* | *ENSAMXG00000013071* | *Ensemble* |
| *C1GALT1b* | *Astyanax mexicanus* | *ENSAMXG00000001374* | *Ensemble* |
| *C1GALT1a* | *Oreochromis niloticus* | *ENSONIG00000012108* | *Ensemble* |
| *C1GALT1b* | *Oreochromis niloticus* | *ENSONIG00000019092* | *Ensemble* |
| *C1GALT1a* | *Oryzias latipes* | *ENSORLG00000007498* | *Ensemble* |
| *C1GALT1b* | *Oryzas latipes* | *ENSORLG00000001643* | *Ensemble* |
| *C1GALT1a* | *Takifugu rubripes* | *ENSTRUG00000013143* | *Ensemble* |
| *C1GALT1b* | *Takifugu rubripes* | *ENSTRUG00000013063* | *Ensemble* |
| *C1GALT1a* | *Tetraodon nigroviridis* | *ENSTNIG00000015077* | *Ensemble* |
| *C1GALT1b* | *Tetraodon nigroviridis* | *ENSTNIG00000014583* | *Ensemble* |
| *C1GALT1a* | *Gasterosteus aculeatus* | *ENSGACG00000008969* | *Ensemble* |
| *C1GALT1b* | *Gasterosteus aculeatus* | *ENSGACG00000000710* | *Ensemble* |
| *C1GALT1a* | *Poecilia formosa* | *ENSPFOG00000008682* | *Ensemble* |
| *C1GALT1b* | *Poecilia formosa* | *ENSPFOG00000011251* | *Ensemble* |
| *C1GALT1a* | *Xiphophorus maculatus* | *ENSXMAG00000004968* | *Ensemble* |
| *C1GALT1b* | *Xiphophorus maculatus* | *ENSXMAG00000002052* | *Ensemble* |
| *C1GALT1a* | *Gadus morhua* | *ENSGMOG00000001538* | *Ensemble* |
| *C1GALT1b* | *Gadus morhua* | *ENSGMOG00000007524* | *Ensemble* |
| *C1GALT1* | *Callorhinchus milii* | *XP_007902959.1* | *NCBI* |

**Supplementary Table 5.** Name and accession number of the B4GALNT1 amino-acid sequences used in the alignment (Fig S4) and phylogeny analysis (Fig S5).

| Pre-pro-peptide | Species | Accession number | Annotation provider |
|---|---|---|---|
| *B4GALNT1* | *Latimeria chalumnae* | *ENSLACG00000018149* | *Ensembl* |
| *B4GALNT1* | *Xenopus tropicalis* | *NP_001120168.1* | *NCBI* |
| *B4GALNT1* | *Anolis carolensis* | *ENSACAG00000007194* | *Ensembl* |
| *B4GALNT1* | *Alligator mississippiensis* | *XP_014464072.1* | *NCBI* |
| *B4GALNT1* | *Ovis aries* | *ENSOARP00000006086* | *Ensembl* |
| *B4GALNT1* | *Homo sapiens* | *ENSG00000135454* | *Ensembl* |
| *B4GALNT1* | *Lepisosteus oculatus* | *ENSLOCG00000007549* | *Ensembl* |
| *B4GALNT1a* | *Anguilla anguilla* | *DAA_B4GN1.4.4* | *Phylofish* |
| *B4GALNT1b* | *Anguilla anguilla* | *DAA_B4GN1.1.4* | *Phylofish* |
| *B4GALNT1a* | *Scleropages formosus* | *XM_018760130.1* | *NCBI* |
| *B4GALNT1b* | *Scleropages formosus* | *XM_018726028.1* | *NCBI* |
| *B4GALNT1a* | *Danio rerio* | *ENSDARG00000061520* | *Ensembl* |
| *B4GALNT1b* | *Danio rerio* | *ENSDARG00000077352* | *Ensembl* |
| *B4GALNT1a* | *Astyanax mexicanus* | *ENSAMXG00000001403* | *Ensembl* |
| *B4GALNT1b* | *Astyanax mexicanus* | *TAM_LOC793635.2.2* | *Phylofish* |
| *B4GALNT1a* | *Oreochromis niloticus* | *ENSONIG00000012109* | *Ensembl* |
| *B4GALNT1b* | *Oreochromis niloticus* | *ENSONIG00000019085* | *Ensembl* |
| *B4GALNT1a* | *Oryzias latipes* | *XM_020704642.1* | *NCBI* |
| *B4GALNT1b* | *Oryzias latipes* | *ENSORLG00000001618* | *Ensembl* |
| *B4GALNT1a* | *Takifugu rubripes* | *Predicted* | |
| *B4GALNT1a* | *Tetraodon nigroviridis* | *Predicted* | |
| *B4GALNT1b1* | *Tetraodon nigroviridis* | *ENSTNIG00000014585* | *Ensembl* |
| *B4GALNT1a* | *Gasterosteus aculeatus* | *Predicted* | |
| *B4GALNT1b* | *Gasterosteus aculeatus* | *ENSGACG00000000713* | *Ensembl* |
| *B4GALNT1a1* | *Poecilia formosa* | *ENSPFOG00000008471* | *Ensembl* |
| *B4GALNT1a2* | *Poecilia formosa* | *ENSPFOG00000000900* | *Ensembl* |
| *B4GALNT1b1* | *Poecilia formosa* | *ENSPFOG00000011391* | *Ensembl* |
| *B4GALNT1a* | *Xiphophorus maculatus* | *XM_023344035.1* | *NCBI* |
| *B4GALNT1b1* | *Xiphophorus maculatus* | *ENSXMAG00000002029* | *Ensembl* |
| *B4GALNT1b2* | *Xiphophorus maculatus* | *ENSXMAG00000001838* | *Ensembl* |
| *B4GALNT1* | *Callorhinchus milii* | *XM_007909947.1* | *NCBI* |
